# Numerical investigation of two-dimensional fuzzy fractional heat problem with an external source variable

**DOI:** 10.1371/journal.pone.0304871

**Published:** 2024-06-21

**Authors:** Muhammad Nadeem, Saad H. Alotaibi, Fawziah M. Alotaibi, Yahya Alsayaad

**Affiliations:** 1 School of Mathematics and Statistics, Qujing Normal University, Qujing, China; 2 Department of Chemistry, Turabah University College, Taif University, Taif, Saudi Arabia; 3 Department of Mathematics, Turabah University College, Taif University, Taif, Saudi Arabia; 4 Department of Physics, Hodeidah University, Al-Hudaydah, Yemen; University of Porto Faculty of Engineering: Universidade do Porto Faculdade de Engenharia, PORTUGAL

## Abstract

This study suggests a strategy for calculating the fuzzy analytical solutions to a two-dimensional fuzzy fractional-order heat problem including a diffusion variable connected externally. We propose Sawi residual power series scheme (SRPSS) which is the amalgamation of Sawi transform and residual power series scheme under the Caputo fractional differential operator. We demonstrate three different examples to derive the fuzzy fractional series solution which is characterized by its rapid convergence and easy finding of the unknown coefficients using the concept of limit at infinity. The most significant aspect of this scheme is that it derives the results without time effort compared with the traditional residual power series approach. Our findings confirm that the SRPSS is a robust and valuable method for approximating the solution of fuzzy fractional problems. Furthermore, we provide 2D and 3D symbolic representations to present the physical behavior of fuzzy fractional problems under the lower and upper bounded solutions.

## 1 Introduction

Many real-life events in physics, chemistry, control theory, and other scientific and engineering domains can be lucratively modeled using fractional derivatives [1–3]. The most important reason is that fractional calculus allows for the appropriate modeling of real-world problems, which in turn depends on both the current and the preceding chronological period. The fractional differential equations (FDEs) have garnered attention from many scientific and engineering academics for their crucial role in comprehending various real-life processes in the natural sciences [4, 5]. These processes include mechanical systems, wave propagation phenomena, earthquake modeling, image processing, and control theory. Fractional calculus can be employed to characterize and reframe these processes as FDEs. The utilization of FDEs in the aforesaid and other phenomena is remarkable due to their characteristic of non-locality. In the same vein, the differential operators provide an excellent framework for describing the memory and genetic features of several processes and substances.

The theory of fuzzy sets was proposed by Zadeh [6] in 1965 as a natural progression from classical set theory. Therefore, many researchers have started to take an interest because of its proficiency in studying the unpredictability of facts and specifics. Fuzzy calculus has become highly important in a wide range of disciplines, including topology, fixed-point principle, integral inequalities, fractional algebra, bifurcations, image processing, recognition of patterns, intelligent systems, electronic devices, theory of control, and machine learning [7–9]. Fuzzy set theory primarily enables us to generate new estimates and enhance the opportunities for efficiently managing and analyzing imprecise information. Several scholars expanded on this idea to construct primary fuzzy calculus based on fuzzy mapping and control. Fuzzy calculus involves the study of fuzzy sets and fuzzy numbers, which enable the representation and manipulation of uncertain and imprecise numbers. Thus, numerous scholars have studied FDEs [10–12].

Consider the fuzzy fractional heat problem in two dimensions, such as

$$\begin{array}{c} D_{\tau }^{\alpha }\tilde{\vartheta }\left(\varsigma ,\mu ,\tau \right)=D_{\varsigma }^{2}\tilde{\vartheta }\left(\varsigma ,\mu ,\tau \right)+D_{\mu }^{2}\tilde{\vartheta }\left(\varsigma ,\mu ,\tau \right)+\tilde{g}\left(\varsigma ,\mu ,\tau \right), \end{array}$$ (1)

with initial condition

$$\begin{array}{c} \tilde{\vartheta }\left(\varsigma ,\mu ,0\right)=\tilde{f}\left(\varsigma ,\mu \right), \end{array}$$ (2)

where *α* represents the Caputo fractional derivative and $g\in C\left(\left[0,\infty \right)\times \left[0,\infty \right)\times \left[0,\infty \right),\left[0,\infty \right)\right),\tilde{f}\in \left(\left[0,\infty \right)\times \left[0,\infty \right),\left[0,\infty \right)\right)$. The 2D heat problem is defined as the heat transfer occurring through an infinitely thin substance. In Eq (1), the parameter $“\tilde{\vartheta }”$ shows the temperature of an object at any stage of the thin substance. This phenomenon of heat flow can be studied in numerous areas of physical science and technology. Hence, the examination of two-dimensional Fuzzy fractional heat equations holds significant relevance in diverse fields, including the analysis of heat transfer in substances with uncertain features, the numerical modeling of variation in temperature in biological structures, and the study of temperature phenomena in intricate systems with fuzzy variables.

Fuzzy fractional differential and integral equations have received significant attention in the physical sciences during the past few decades. Many researchers have obtained the solution of fuzzy differential equations using various analytical and numerical techniques such as homotopy perturbation method [13], Fuzzy Laplace transform method [14], Crank-Nicolson method [15], Natural adomian decomposition method [16], SM’s method [17]. Akram and Bilal [18] utilized homotopy perturbation scheme for the analytical solution of fuzzy heat problem. Allahviranloo [19] applied a finite difference scheme to calculate the numerical solution of fuzzy heat and wave problems. Georgieva [20] utilized double Fuzzy Sumudu transform to solve partial Volterra fuzzy integro-differential equations. Mazandarani and Xiu [21] expanded the application of the differential transformation strategy to deal with fuzzy PDEs using strongly generalized differentiability. This method was discovered to be a direct and effective approach for getting analytical-numerical results. Osman et al. [22] introduced some analytical schemes for obtaining the solution of fuzzy fractional PDEs.

In the present study, we combine the Sawi transform and residual power series scheme to obtain the fuzzy results of fuzzy fractional heat problem in two-dimensional form. In the current framework, noteworthy findings are demonstrated without any restrictions being placed on the variables or assumptions being made regarding the parameters. The obvious benefit of this method is that it displays the outcomes in terms of power series, which enables one to get precise outcomes in a relatively short amount of time. Furthermore, we do not consider He’s polynomials, which could potentially make the actual problems more complicated. Some graphical structures are presented for each problem, with different fractional order. The rest of paper is designed as follows: Section (2) consists of fuzzy definitions and the basic concept of Sawi transformation. We formulate the strategy of SRPSS in Sections (3) for fuzzy fractional problems. In Sections (4), we show the authenticity and reliability of SRPSS by considering three numerical applications in Section (4). The conclusion is summarized in Section (5).

## 2 Fuzzy integral and Sawi transform

Some concepts of fuzzy integrals and Sawi transform are presented in this section.

**Definition 2.1** Let *α* > 1, then Riemann-Liouville operator is expressed as [23, 24]

$$\begin{array}{c} J^{\alpha }\vartheta (\varsigma )=\frac{1}{\Gamma (\alpha )}\int_{0}^{\varsigma }{\left(\varsigma -\tau \right)}^{\alpha -1}\vartheta (\tau )d\tau ,(\alpha >0)\\ J^{0}\vartheta (\varsigma )=\vartheta (\varsigma ). \end{array}$$

Also, we have

$$\begin{array}{c} J^{\alpha }\varsigma^{q}=\frac{\Gamma (q+1)}{\Gamma (q+\alpha +1)}\varsigma^{\alpha +q}. \end{array}$$

**Definition 2.2** The Caputo fractional derivative is [25]:

$$\begin{array}{c} D_{\varsigma }^{\alpha }\vartheta \left(\varsigma \right)=J^{k-\alpha }D^{n}f\left(\tau \right)=\frac{1}{\Gamma (n-\alpha )}\int_{0}^{\varsigma }{\left(\varsigma -\xi \right)}^{k-\alpha -1}\vartheta^{k}\left(\xi \right)d\xi , \end{array}$$

for k-1<α≤k,k∈N,τ>0.

**Definition 2.3** The Sawi transform is defined as [26]

$$\begin{array}{c} S\left[\vartheta \left(\tau \right)\right]=R\left(\omega \right)=\frac{1}{\omega^{2}}\int_{0}^{\infty }\vartheta \left(\tau \right)e^{-\frac{\tau }{\omega }}d\tau ,\;\;\;\tau \geq 0,\;\;\;\omega \geq 0, \end{array}$$

where *ω* shows the transformation variable. If *ϑ*(℘) is exponentially ordered and piecewise continuous, then the ST of the function *ϑ*(*τ*), *τ* ≥ 0 exists; otherwise, ST might not exist. Let we have *R*(*ω*) as ST of *ϑ*(*τ*), therefore *ϑ*(*τ*) be the inverse of *R*(*ω*) as follows,

$$\begin{array}{c} S^{-1}\left[R\left(\omega \right)\right]=\vartheta \left(\tau \right),\;\;\;\;\;S^{-1}\;\text{be}\;\text{inverse}\;\text{ST}. \end{array}$$

**Definition 2.4** If *S*{*ϑ*_1_(*τ*)} = *ς*_1_(*ω*) and *S*{*ϑ*_2_(*τ*)} = *ς*_2_(*ω*), then [27, 28]

$$\begin{array}{c} S\left\{a_{1}\vartheta_{1}\left(\tau \right)+a_{2}\vartheta_{2}\left(\tau \right)\right\}=S\left\{a_{1}\vartheta_{1}\left(\tau \right)\right\}+a_{2}S\left\{\vartheta_{2}\left(\tau \right)\right\}, \end{array}$$

that yields the linear property as

$$\begin{array}{c} S\left\{a_{1}\vartheta_{1}\left(\tau \right)+ba_{2}\vartheta_{2}\left(\tau \right)\right\}=a\varsigma_{1}\left(\tau \right)+b\varsigma_{2}\left(\tau \right). \end{array}$$

where *a* and *b* are unknown numbers.

**Proposition 1** As we have, *S*{*ϑ*(*τ*)} = *R*(*ω*), thus we can state the following properties as

*a)* $S\left\{\vartheta^{\prime }\left(\tau \right)\right\}=\frac{R(\omega )}{\omega }-\frac{\vartheta (0)}{\omega^{2}},$

*b)* $S\left\{\vartheta^{\prime \prime }\left(\tau \right)\right\}=\frac{R(\omega )}{\omega^{2}}-\frac{\vartheta (0)}{\omega^{3}}-\frac{\vartheta^{\prime }\left(0\right)}{\omega^{2}},$

*c)* $S\left\{\vartheta^{m}\left(\tau \right)\right\}=\frac{R(\omega )}{\omega^{m}}-\frac{\vartheta (0)}{\omega^{m+1}}-\frac{\vartheta^{\prime }\left(0\right)}{\omega^{m}}-\cdots -\frac{\vartheta^{m-1}\left(0\right)}{\omega^{2}}.$

**Definition 2.5** The derivative of the function *ϑ*(*τ*) with respect to the variable *τ* is referred to as the ST of Caputo as follows [29]

$$\begin{array}{c} S\left[D_{\tau }^{\alpha }\vartheta \left(\tau \right)\right]=\frac{1}{\omega^{\alpha }}S\left[\vartheta \left(\tau \right)\right]-\sum_{k=0}^{n-1}{\left(\frac{1}{\omega }\right)}^{\alpha -k+1}\vartheta^{k}\left(0\right). \end{array}$$

**Definition 2.6** Suppose a series such as [30]

$$\begin{array}{c} \sum_{n=0}^{\infty }\vartheta_{n}{\left(\tau -\tau_{0}\right)}^{n\alpha }=\vartheta_{0}\left(\varsigma \right)+\vartheta_{1}\left(\varsigma \right){\left(\tau -\tau_{0}\right)}^{\alpha }+\vartheta_{2}\left(\varsigma \right){\left(\tau -\tau_{0}\right)}^{2\alpha }+\cdots ,\;0<n-1<\alpha \leq n,\;\tau \geq \tau_{0}. \end{array}$$

This formulation can be represented as a power series centered at *τ* = *τ*_0_, where *τ* is the variable and *ς* represents the coefficients of the series.

**Definition 2.7** Suppose $\tilde{\vartheta }$ is a subset of the region *δ* and is a continuous fuzzy parameter defined on the interval [0, *b*]. The Riemann-Liouville theory defines the fractional integral connected with *δ* as follows [31]

$$\begin{array}{c} I^{\eta }\tilde{\vartheta }\left(\delta \right)=\int_{0}^{\delta }\frac{{\left(\delta -\varepsilon \right)}^{\eta -1}\tilde{\vartheta }\left(\varepsilon \right)}{\Gamma (\eta )}d\eta ,\;\varepsilon \in \left(0,\infty \right). \end{array}$$

Further, if $\tilde{\vartheta }\in C^{F}\left[0,b\right]\cap L^{F}\left[0,b\right]$ then *C*^*F*^[0, *b*] and *L*^*F*^[0, *b*] shows the is fuzzy continues and fuzzy Lebesgue integrable functions accordingly, thus the fuzzy fractional integral is stated as

$$\begin{array}{c} {\left[I^{\eta }\tilde{\vartheta }\left(\delta \right)\right]}_{\delta }=[I^{\eta }{\underline{\vartheta }}_{\delta }\left(\delta \right),I^{\eta }{\bar{\vartheta }}_{\delta }\left(\delta \right)],\;0\leq \delta \leq 1 \end{array}$$

thus

$$\begin{array}{c} \begin{array}{cc} & I^{\eta }{\underline{\vartheta }}_{\delta }\left(\delta \right)=\int_{0}^{\delta }\frac{{\left(\delta -\varepsilon \right)}^{\eta -1}{\underline{\vartheta }}_{\delta }\left(\varepsilon \right)}{\Gamma (\eta )}d\varepsilon ,\;\eta ,\varepsilon \in \left(0,\infty \right),\\ & I^{\eta }{\bar{\vartheta }}_{\delta }\left(\delta \right)=\int_{0}^{\delta }\frac{{\left(\delta -\varepsilon \right)}^{\eta -1}{\bar{\vartheta }}_{\delta }\left(\varepsilon \right)}{\Gamma (\eta )}d\varepsilon ,\;\eta ,\varepsilon \in \left(0,\infty \right). \end{array} \end{array}$$

**Definition 2.8** Let $\tilde{\vartheta }\in C^{F}\left[0,b\right]\cap L^{F}\left[0,b\right]$ so that $[{\underline{\vartheta }}_{\delta }\left(\tau \right),{\bar{\vartheta }}_{\delta }\left(\tau \right)],\delta \in \left[0,1\right]$ and *τ*_0_ ∈ (0, *b*), thus, the fuzzy Caputo fractional derivative is defined as [31]

$$\begin{array}{c} {\left[D^{\eta }\tilde{\vartheta }(\tau_{0})\right]}_{\delta }=[D^{\eta }{\underline{\vartheta }}_{\delta }(\tau_{0}),D^{\eta }{\bar{\vartheta }}_{\delta }(\tau_{0})],\;0<\eta \leq 1, \end{array}$$

where

$$\begin{array}{c} \begin{array}{cc} & D^{\eta }{\underline{\vartheta }}_{\delta }(\tau_{0})={\left[\int_{0}^{\tau }\frac{{\left(\tau -\varepsilon \right)}^{m-\eta -1}\frac{d^{m}}{d\varepsilon^{m}}\vartheta_{\delta }\left(\varepsilon \right)}{\Gamma (m-\eta )}d\varepsilon \right]}_{\tau =\tau_{0}},\\ & D^{\eta }{\bar{\vartheta }}_{\delta }(\tau_{0})={\left[\int_{0}^{\tau }\frac{{\left(\tau -\varepsilon \right)}^{m-\eta -1}\frac{d^{m}}{d\varepsilon^{m}}{\bar{\vartheta }}_{\delta }\left(\varepsilon \right)}{\Gamma (m-\eta )}d\varepsilon \right]}_{\tau =\tau_{0}}, \end{array} \end{array}$$

such that the integral on the right side converges and *m* = ⌈*η*⌉. Since *η* ∈ (0, 1] so *m* = 1.

**Definition 2.9** The lower and upper boundaries of all fuzzy elements should meet these constraints. [32]

- (i) The function $\underline{f}\left(\delta \right)$ is non-reducing and constantly left on the time frame [0, 1].
- (ii) The function $\bar{f}\left(\delta \right)$ is a non-reducing and constantly right on the time frame [0, 1].
- (iii) $\underline{f}\left(\delta \right)\leq \bar{f}\left(\delta \right),\;0\leq \delta \leq 1$.

Let $\underline{f}\left(\delta \right)=\bar{f}\left(\delta \right)=\delta$, so *δ* shows a crisp element.

## 3 Construction of Sawi residual power series scheme (SRPSS)

This section introduces the Sawi residual power series scheme, which is utilized to approximate the solutions of the two-dimensional fuzzy fractional heat problem. This technique can be constructed directly without taking into account any assumptions. Let’s assume the following nonlinear fuzzy fractional problem

$$\begin{array}{c} D_{\tau }^{\alpha }\tilde{\vartheta }\left(\varsigma ,\mu ,\tau \right)=L\tilde{\vartheta }\left(\varsigma ,\mu ,\tau \right)+N\tilde{\vartheta }\left(\varsigma ,\mu ,\tau \right)+\tilde{g}\left(\varsigma ,\mu ,\tau \right), \end{array}$$ (3)

with following constraints

$$\begin{array}{c} \tilde{\vartheta }\left(\varsigma ,\mu ,0\right)=\tilde{f}\left(\varsigma ,\mu \right). \end{array}$$ (4)

**Step 1:** By using *S*T to Eq (3), it gives

$$\begin{array}{c} S[D_{\tau }^{\alpha }\tilde{\vartheta }\left(\varsigma ,\mu ,\tau \right)]=S[L\tilde{\vartheta }\left(\varsigma ,\mu ,\tau \right)+N\tilde{\vartheta }\left(\varsigma ,\mu ,\tau \right)+\tilde{g}\left(\varsigma ,\mu ,\tau \right)]. \end{array}$$

By applying the definition of *S*T with constraints (4), we obtain

$$\begin{array}{c} \frac{1}{\omega^{\alpha }}S\left[\tilde{\vartheta }\left(\varsigma ,\mu ,\tau \right)\right]-\frac{1}{\omega^{\alpha +1}}\tilde{\vartheta }\left(\varsigma ,\mu ,0\right)=S[L\tilde{\vartheta }\left(\varsigma ,\mu ,\tau \right)+N\tilde{\vartheta }\left(\varsigma ,\mu ,\tau \right)+\tilde{g}\left(\varsigma ,\mu ,\tau \right)]. \end{array}$$

or we may write it as

$$\begin{array}{c} S\left[\tilde{\vartheta }\left(\varsigma ,\mu ,\tau \right)\right]=\frac{1}{\omega }\tilde{\vartheta }\left(\varsigma ,\mu ,0\right)+\omega^{\alpha }S[L\tilde{\vartheta }\left(\varsigma ,\mu ,\tau \right)+N\tilde{\vartheta }\left(\varsigma ,\mu ,\tau \right)+\tilde{g}\left(\varsigma ,\mu ,\tau \right)]. \end{array}$$ (5)

**Step 2:** Applying inverse *S*T to Eq 5, we get

$$\begin{array}{c} \tilde{\vartheta }\left(\varsigma ,\mu ,\tau \right)=\tilde{G}\left(\varsigma ,\mu ,\tau \right)+S^{-1}[\omega^{\alpha }\{L\tilde{\vartheta }\left(\varsigma ,\mu ,\tau \right)+N\tilde{\vartheta }\left(\varsigma ,\mu ,\tau \right)\}], \end{array}$$ (6)

with

$$\begin{array}{c} \tilde{G}\left(\varsigma ,\tau \right)=\tilde{f}\left(\varsigma ,\mu \right)+S^{-1}[\omega^{\alpha }\{\tilde{g}\left(\varsigma ,\mu ,\tau \right)\}]. \end{array}$$

**Step 3:** Let the general solution of Eq (3) can be written as:

$$\begin{array}{c} \tilde{\vartheta }\left(\varsigma ,\mu ,\tau \right)=\sum_{n=0}^{\infty }\;{\tilde{p}}_{n}\left(\varsigma ,\mu \right)\frac{\tau^{n\alpha }}{\Gamma (n\alpha +1)}. \end{array}$$ (7)

Thus, the truncated series results is

$$\begin{array}{c} {\tilde{\vartheta }}_{k}\left(\varsigma ,\mu ,\tau \right)=\sum_{n=0}^{k}\;{\tilde{p}}_{n}\left(\varsigma ,\mu \right)\frac{\tau^{n\alpha }}{\Gamma (n\alpha +1)}. \end{array}$$ (8)

**Step 4:** Suppose that $Res_{\tilde{\vartheta }}$ be residual function for Eq (8) is

$$\begin{array}{c} Res_{\tilde{\vartheta }}=\tilde{\vartheta }\left(\varsigma ,\mu ,\tau \right)-[\tilde{G}\left(\varsigma ,\mu ,\tau \right)+S^{-1}\{\omega^{\alpha }(L\tilde{\vartheta }\left(\varsigma ,\mu ,\tau \right)+N\tilde{\vartheta }\left(\varsigma ,\mu ,\tau \right))\}]. \end{array}$$ (9)

Then its truncated residual function yields as

$$\begin{array}{c} Res_{{\tilde{\vartheta }}_{k}}={\tilde{\vartheta }}_{k}\left(\varsigma ,\mu ,\tau \right)-[\tilde{G}\left(\varsigma ,\mu ,\tau \right)+S^{-1}\{\omega^{\alpha }(L{\tilde{\vartheta }}_{k}\left(\varsigma ,\mu ,\tau \right)+N{\tilde{\vartheta }}_{k}\left(\varsigma ,\mu ,\tau \right))\}]. \end{array}$$ (10)

Now, the components of ${\tilde{p}}_{n}\left(\varsigma \right)$ in Eq 8 can be obtained under the following condition:

-
$\;\underset{k\to \infty }{\text{lim}\;}\text{Res}\;{\tilde{\vartheta }}_{k}\left(\varsigma ,\mu ,\tau \right)=\text{Res}\;\tilde{\vartheta }\left(\varsigma ,\mu ,\tau \right)=0$ ,
-
$\;D_{\tau }^{n\alpha }\text{Res}\;{\tilde{\vartheta }}_{k}\left(\varsigma ,\mu ,0\right)=0,\;n=0,1,2,\ldots$

We can achieve the following results:

$$\begin{array}{cc} \tilde{\vartheta }\left(\varsigma ,\mu ,\tau \right) & =\underset{N\to 0}{\text{lim}\;}\sum_{n=0}^{N}{\tilde{p}}_{n}\left(\varsigma ,\mu ,\tau \right), \end{array}$$

with

$$\begin{array}{cc} {\tilde{\vartheta }}_{0}\left(\varsigma ,\mu ,\tau \right) & =\tilde{\vartheta }\left(\varsigma ,\mu ,0\right),\\ {\tilde{\vartheta }}_{1}\left(\varsigma ,\mu ,\tau \right) & ={\tilde{p}}_{1}\left(\varsigma ,\mu \right)\frac{\tau^{\alpha }}{\Gamma (1+\alpha )},\\ {\tilde{\vartheta }}_{2}\left(\varsigma ,\mu ,\tau \right) & ={\tilde{p}}_{2}\left(\varsigma ,\mu \right)\frac{\tau^{2\alpha }}{\Gamma (1+2\alpha )},\\ & \vdots \\ {\tilde{\vartheta }}_{n}\left(\varsigma ,\mu ,\tau \right) & ={\tilde{p}}_{n}\left(\varsigma ,\mu \right)\frac{\tau^{n\alpha }}{\Gamma (1+n\alpha )}. \end{array}$$

Hence, the approximate results derived from Sawi residual power series scheme become as

$$\begin{array}{c} \tilde{\vartheta }\left(\varsigma ,\mu ,\tau \right)={\tilde{\vartheta }}_{0}\left(\varsigma ,\mu ,\tau \right)+{\tilde{\vartheta }}_{1}\left(\varsigma ,\mu ,\tau \right)+{\tilde{\vartheta }}_{2}\left(\varsigma ,\mu ,\tau \right)+{\tilde{\vartheta }}_{3}\left(\varsigma ,\mu ,\tau \right)+\cdots . \end{array}$$

## 4 Numerical applications

In this section, we provide the performance of SRPSS to calculate the fuzzy results of fuzzy heat problems in a two-dimensional form of fractional order. We consider three examples and derive the lower and upper bound solution at multiple fractional order of *α*. The results are shown in terms of fractional power series yielding the convergent solution just after a small number of repetitions. All the results are calculated with the help of Mathematica software.

### 4.1 Example 1

Suppose the following 2D fuzzy heat problem of fractional order as follows

$$\begin{array}{c} D_{\tau }^{\alpha }\tilde{\vartheta }\left(\varsigma ,\mu ,\tau \right)={\tilde{\vartheta }}_{\varsigma \varsigma }\left(\varsigma ,\mu ,\tau \right)+{\tilde{\vartheta }}_{\mu \mu }\left(\varsigma ,\mu ,\tau \right)+\varsigma +\mu +\tau , \end{array}$$ (11)

with following constraints

$$\begin{array}{c} \tilde{\vartheta }\left(\varsigma ,\mu ,0\right)=\tilde{f}e^{-(\varsigma +\mu )}. \end{array}$$ (12)

with $\tilde{f}=\left[\underline{f},\bar{f}\right]=\left[\delta -1,1-\delta \right]$.

#### 4.1.1 Lower bound findings for 2D fuzzy problem

The lower bound fuzzy problem can be derived from Eq (11) as follows

$$\begin{array}{c} \frac{\partial^{\alpha }\underline{\vartheta }}{\partial \tau^{\alpha }}=\frac{\partial^{2}\underline{\vartheta }}{\partial \varsigma^{2}}+\frac{\partial^{2}\underline{\vartheta }}{\partial \mu^{2}}+\varsigma +\mu +\tau , \end{array}$$ (13)

with lower fuzzy initial constraints

$$\begin{array}{c} \underline{\vartheta_{0}}\left(\varsigma ,\mu ,\tau \right)=\left(\delta -1\right)e^{-(\varsigma +\mu )}. \end{array}$$ (14)

Making the use of ST, we get

$$\begin{array}{c} S[\frac{\partial^{\alpha }\underline{\vartheta }}{\partial \tau^{\alpha }}]=S[\frac{\partial^{2}\underline{\vartheta }}{\partial \varsigma^{2}}+\frac{\partial^{2}\underline{\vartheta }}{\partial \mu^{2}}+\varsigma +\mu +\tau ]. \end{array}$$

Using the definition of ST in the sense of Caputo fractional order, we obtain

$$\begin{array}{c} S\left[\underline{\vartheta }\left(\varsigma ,\mu ,\tau \right)\right]=\frac{1}{\omega }\underline{\vartheta_{0}}\left(\varsigma ,\mu ,\tau \right)+\omega^{\alpha }S[\frac{\partial^{2}\underline{\vartheta }}{\partial \varsigma^{2}}+\frac{\partial^{2}\underline{\vartheta }}{\partial \mu^{2}}+\varsigma +\mu +\tau ]. \end{array}$$

By the use of inverse *S*T, it gives

$$\begin{array}{c} \tilde{\vartheta }\left(\varsigma ,\mu ,\tau \right)=\underline{\vartheta_{0}}\left(\varsigma ,\mu ,\tau \right)+S^{-1}[\omega^{\alpha }\{S(\frac{\partial^{2}\underline{\vartheta }}{\partial \varsigma^{2}}+\frac{\partial^{2}\underline{\vartheta }}{\partial \mu^{2}}+\varsigma +\mu +\tau )\}]. \end{array}$$

By employing the SRPSS, we obtain the following results:

$$\begin{array}{cc} \underline{\vartheta_{1}}\left(\varsigma ,\mu ,\tau \right) & =2\left(\delta -1\right)e^{-(\varsigma +\mu )}\frac{\tau^{\alpha }}{\Gamma (\alpha +1)},\\ \underline{\vartheta_{2}}\left(\varsigma ,\mu ,\tau \right) & =4\left(\delta -1\right)e^{-(\varsigma +\mu )}\frac{\tau^{2\alpha }}{\Gamma (2\alpha +1)},\\ \underline{\vartheta_{3}}\left(\varsigma ,\mu ,\tau \right) & =8\left(\delta -1\right)e^{-(\varsigma +\mu )}\frac{\tau^{3\alpha }}{\Gamma (3\alpha +1)},\\ & \vdots . \end{array}$$

Thus, we can arrange it to the corresponding sequence

$$\begin{array}{c} \begin{array}{cc} \underline{\vartheta }\left(\varsigma ,\mu ,\tau \right) & =\underline{\vartheta_{0}}\left(\varsigma ,\mu ,\tau \right)+\underline{\vartheta_{1}}\left(\varsigma ,\mu ,\tau \right)+\underline{\vartheta_{2}}\left(\varsigma ,\mu ,\tau \right)+\underline{\vartheta_{3}}\left(\varsigma ,\mu ,\tau \right)+\cdots ,\\ & =\left(\delta -1\right)e^{-(\varsigma +\mu )}+\varsigma \frac{\tau^{\alpha }}{\Gamma (\alpha +1)}+\delta \frac{\tau^{\alpha }}{\Gamma (\alpha +1)}+\frac{\tau^{\alpha +1}}{\Gamma (\alpha +2)}+2\left(\delta -1\right)e^{-(\varsigma +\mu )}\frac{\tau^{\alpha }}{\Gamma (\alpha +1)}\\ & +4\left(\delta -1\right)e^{-(\varsigma +\mu )}\frac{\tau^{2\alpha }}{\Gamma (2\alpha +1)}+8\left(\delta -1\right)e^{-(\varsigma +\mu )}\frac{\tau^{3\alpha }}{\Gamma (3\alpha +1)}+\cdots . \end{array} \end{array}$$ (15)

**Note:** If *g*(*ς*, *μ*, *τ*) = 0, then above equation becomes as

$$\begin{array}{c} \begin{array}{cc} \underline{\vartheta }\left(\varsigma ,\mu ,\tau \right) & =\left(\delta -1\right)e^{-(\varsigma +\mu )}+2\left(\delta -1\right)e^{-(\varsigma +\mu )}\frac{\tau^{\alpha }}{\Gamma (\alpha +1)}+4\left(\delta -1\right)e^{-(\varsigma +\mu )}\frac{\tau^{2\alpha }}{\Gamma (2\alpha +1)}\\ & +8\left(\delta -1\right)e^{-(\varsigma +\mu )}\frac{\tau^{3\alpha }}{\Gamma (3\alpha +1)}+\cdots ,\\ & =\left(\delta -1\right)e^{-(\varsigma +\mu )}[1+2\frac{\tau^{\alpha }}{\Gamma (\alpha +1)}+4\frac{\tau^{2\alpha }}{\Gamma (2\alpha +1)}+8\frac{\tau^{3\alpha }}{\Gamma (3\alpha +1)}+\cdots ]. \end{array} \end{array}$$ (16)

This series turns to closed form solutions such as

$$\begin{array}{c} \underline{\vartheta }\left(\varsigma ,\mu ,\tau \right)=\left(\delta -1\right)e^{-(\varsigma +\mu )}\sum_{n=0}^{\infty }\frac{{\left(2\tau^{\alpha }\right)}^{n}}{\Gamma (n\alpha +1)}. \end{array}$$ (17)

#### 4.1.2 Upper bound findings for 2D fuzzy problem

The upper bound fuzzy problem can be derived from Eq (11) as follows

$$\begin{array}{c} \frac{\partial^{\alpha }\bar{\vartheta }}{\partial \tau^{\alpha }}=\frac{\partial^{2}\bar{\vartheta }}{\partial \varsigma^{2}}+\frac{\partial^{2}\bar{\vartheta }}{\partial \mu^{2}}+\varsigma +\mu +\tau , \end{array}$$ (18)

with upper fuzzy initial constraints

$$\begin{array}{c} \bar{\vartheta_{0}}\left(\varsigma ,\mu ,\tau \right)=\left(1-\delta \right)e^{-(\varsigma +\mu )}. \end{array}$$ (19)

By employing the SRPSS, we obtain the following results:

$$\begin{array}{cc} \bar{\vartheta_{1}}\left(\varsigma ,\mu ,\tau \right) & =2\left(1-\delta \right)e^{-(\varsigma +\mu )}\frac{\tau^{\alpha }}{\Gamma (\alpha +1)},\\ \bar{\vartheta_{2}}\left(\varsigma ,\mu ,\tau \right) & =4\left(1-\delta \right)e^{-(\varsigma +\mu )}\frac{\tau^{2\alpha }}{\Gamma (2\alpha +1)},\\ \bar{\vartheta_{3}}\left(\varsigma ,\mu ,\tau \right) & =8\left(1-\delta \right)e^{-(\varsigma +\mu )}\frac{\tau^{3\alpha }}{\Gamma (3\alpha +1)},\\ & \vdots . \end{array}$$

Therefore, we may organize it into the appropriate sequence

$$\begin{array}{c} \begin{array}{cc} \bar{\vartheta }\left(\varsigma ,\mu ,\tau \right) & =\bar{\vartheta_{0}}\left(\varsigma ,\mu ,\tau \right)+\bar{\vartheta_{1}}\left(\varsigma ,\mu ,\tau \right)+\bar{\vartheta_{2}}\left(\varsigma ,\mu ,\tau \right)+\bar{\vartheta_{3}}\left(\varsigma ,\mu ,\tau \right)+\cdots ,\\ & =\left(1-\delta \right)e^{-(\varsigma +\mu )}+\varsigma \frac{\tau^{\alpha }}{\Gamma (\alpha +1)}+\mu \frac{\tau^{\alpha }}{\Gamma (\alpha +1)}+\frac{\tau^{\alpha +1}}{\Gamma (\alpha +2)}+2\left(1-\delta \right)e^{-(\varsigma +\mu )}\frac{\tau^{\alpha }}{\Gamma (\alpha +1)}\\ & +4\left(1-\delta \right)e^{-(\varsigma +\mu )}\frac{\tau^{2\alpha }}{\Gamma (2\alpha +1)}+8\left(1-\delta \right)e^{-(\varsigma +\mu )}\frac{\tau^{3\alpha }}{\Gamma (3\alpha +1)}+\cdots . \end{array} \end{array}$$ (20)

**Note:** When $\tilde{g}\left(\varsigma ,\mu ,\tau \right)=0$, the preceding equation is transformed into

$$\begin{array}{c} \begin{array}{cc} \bar{\vartheta }\left(\varsigma ,\mu ,\tau \right) & =\left(1-\delta \right)e^{-(\varsigma +\mu )}+2\left(1-\delta \right)e^{-(\varsigma +\mu )}\frac{\tau^{\alpha }}{\Gamma (\alpha +1)}+4\left(1-\delta \right)e^{-(\varsigma +\mu )}\frac{\tau^{2\alpha }}{\Gamma (2\alpha +1)}\\ & +8\left(1-\delta \right)e^{-(\varsigma +\mu )}\frac{\tau^{3\alpha }}{\Gamma (3\alpha +1)}+\cdots ,\\ & =\left(1-\delta \right)e^{-(\varsigma +\mu )}[1+2\frac{\tau^{\alpha }}{\Gamma (\alpha +1)}+4\frac{\tau^{2\alpha }}{\Gamma (2\alpha +1)}+8\frac{\tau^{3\alpha }}{\Gamma (3\alpha +1)}+\cdots ]. \end{array} \end{array}$$ (21)

This series turns to closed form solutions such as

$$\begin{array}{c} \bar{\vartheta }\left(\varsigma ,\mu ,\tau \right)=\left(1-\delta \right)e^{-(\varsigma +\mu )}\sum_{n=0}^{\infty }\frac{{\left(2\tau^{\alpha }\right)}^{n}}{\Gamma (n\alpha +1)}. \end{array}$$ (22)

**Fig 1:**
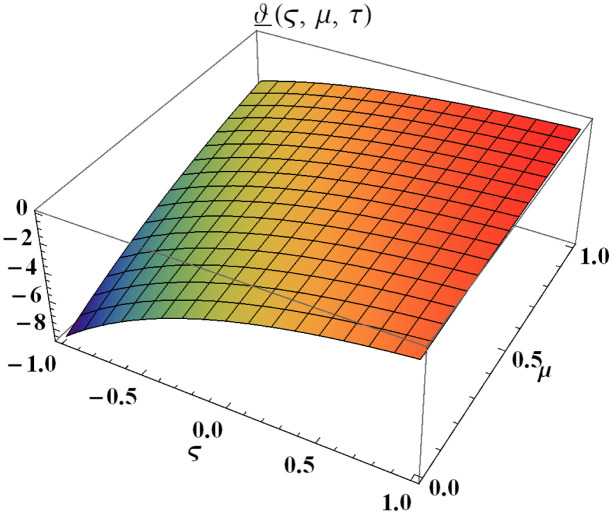
Surface structure of $\underline{\vartheta }\left(\varsigma ,\mu ,\tau \right)$ upon *α* = 0.75.

**Fig 2:**
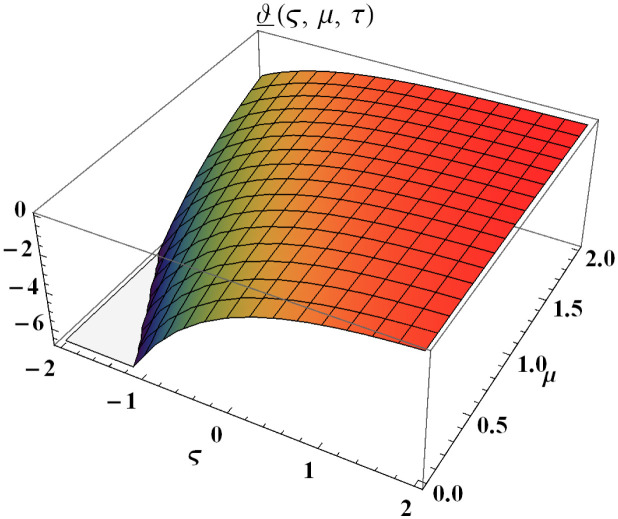
Surface structure of $\underline{\vartheta }\left(\varsigma ,\mu ,\tau \right)$ upon *α* = 1.

**Fig 3:**
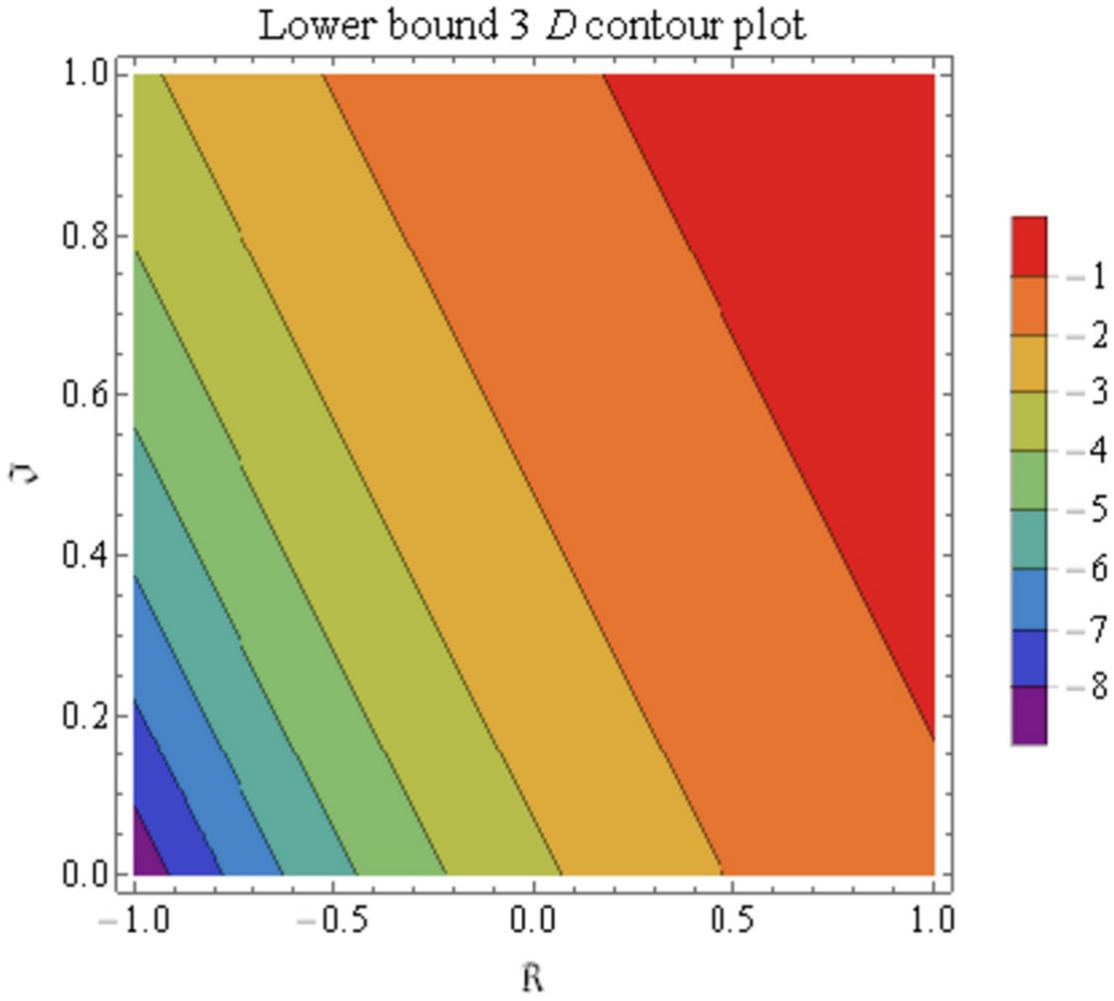
Contour structure of $\underline{\vartheta }\left(\varsigma ,\mu ,\tau \right)$ upon *α* = 0.75.

**Fig 4:**
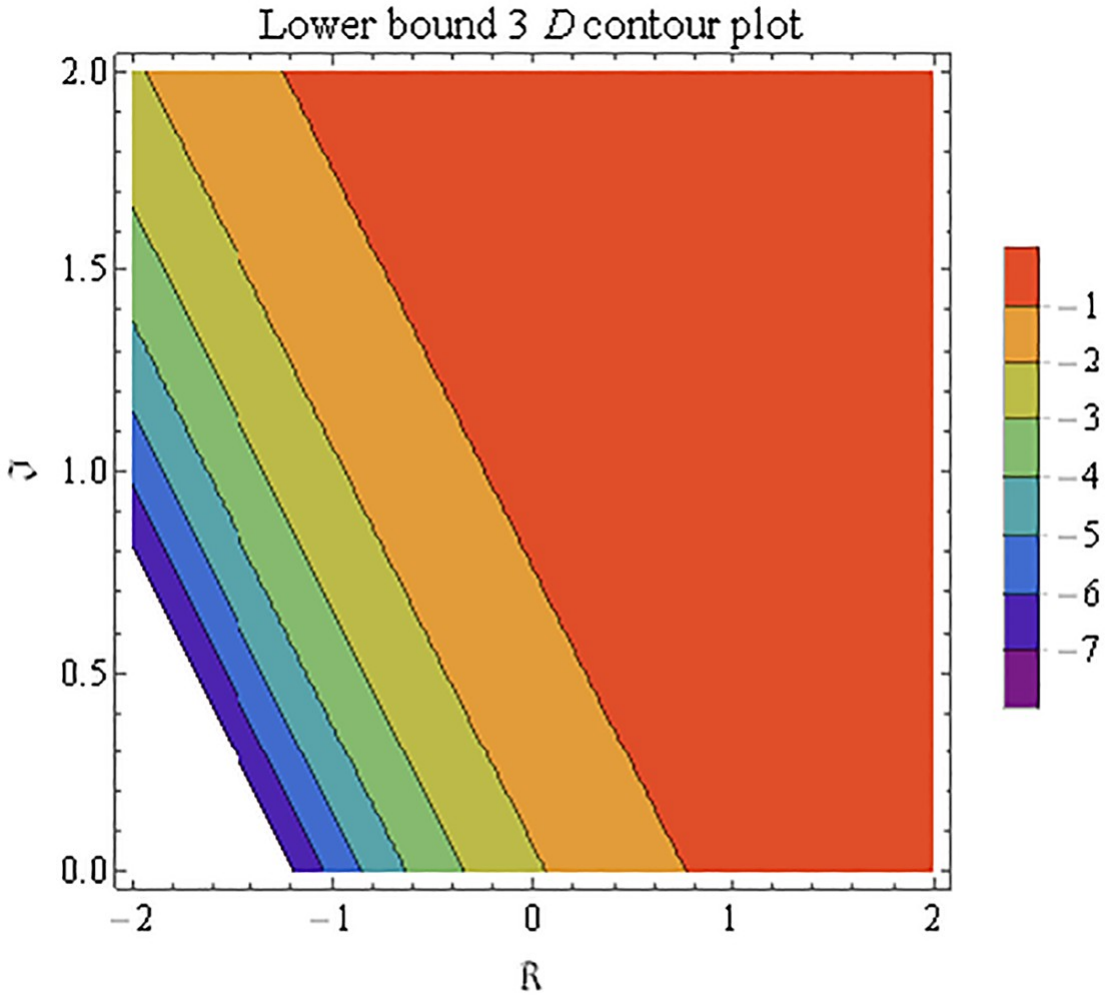
Contour structure of $\underline{\vartheta }\left(\varsigma ,\mu ,\tau \right)$ upon *α* = 1.

**Fig 5:**
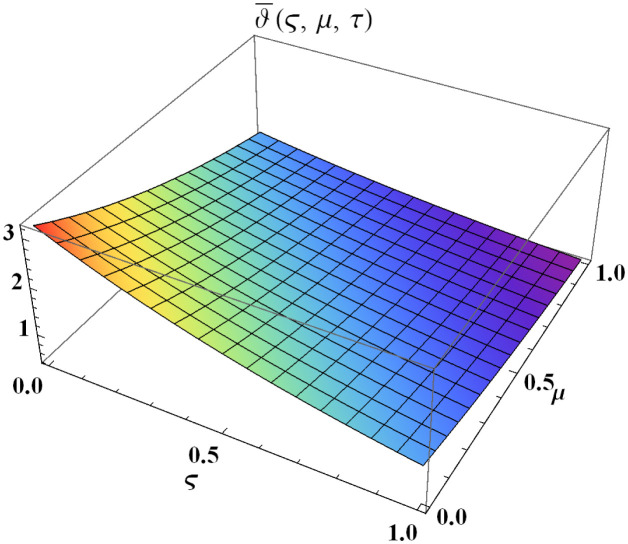
Surface structure of $\bar{\vartheta }\left(\varsigma ,\mu ,\tau \right)$ upon *α* = 0.75.

**Fig 6:**
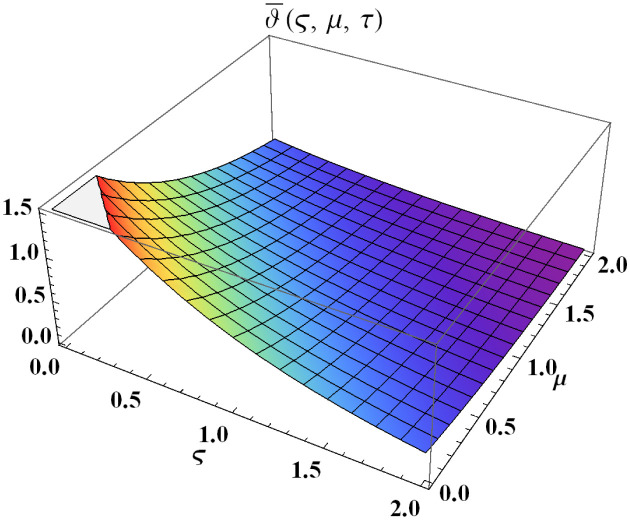
Surface structure of $\bar{\vartheta }\left(\varsigma ,\mu ,\tau \right)$ upon *α* = 1.

**Fig 7:**
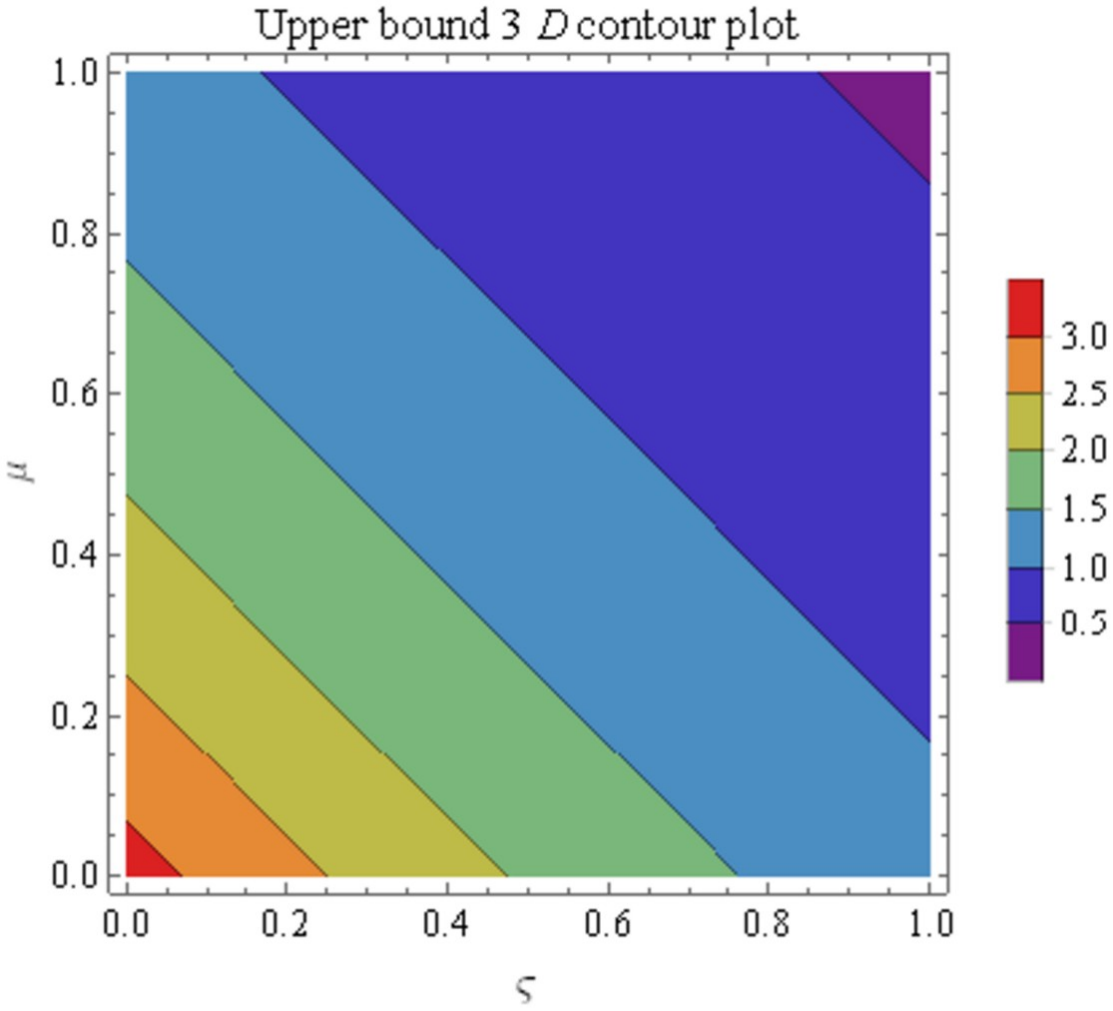
Contour structure of $\bar{\vartheta }\left(\varsigma ,\mu ,\tau \right)$ upon *α* = 0.75.

**Fig 8:**
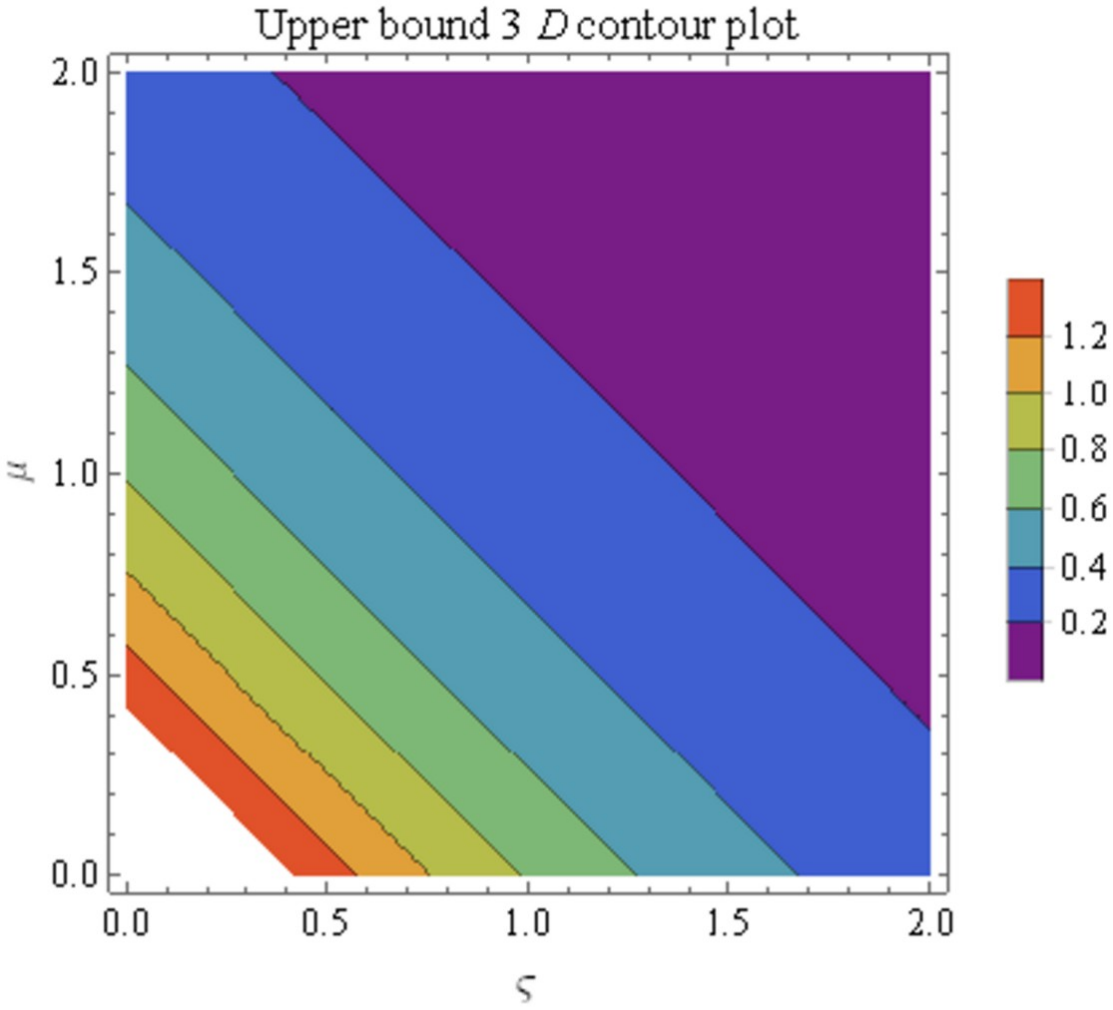
Contour structure of $\bar{\vartheta }\left(\varsigma ,\mu ,\tau \right)$ upon *α* = 1.

**Fig 9:**
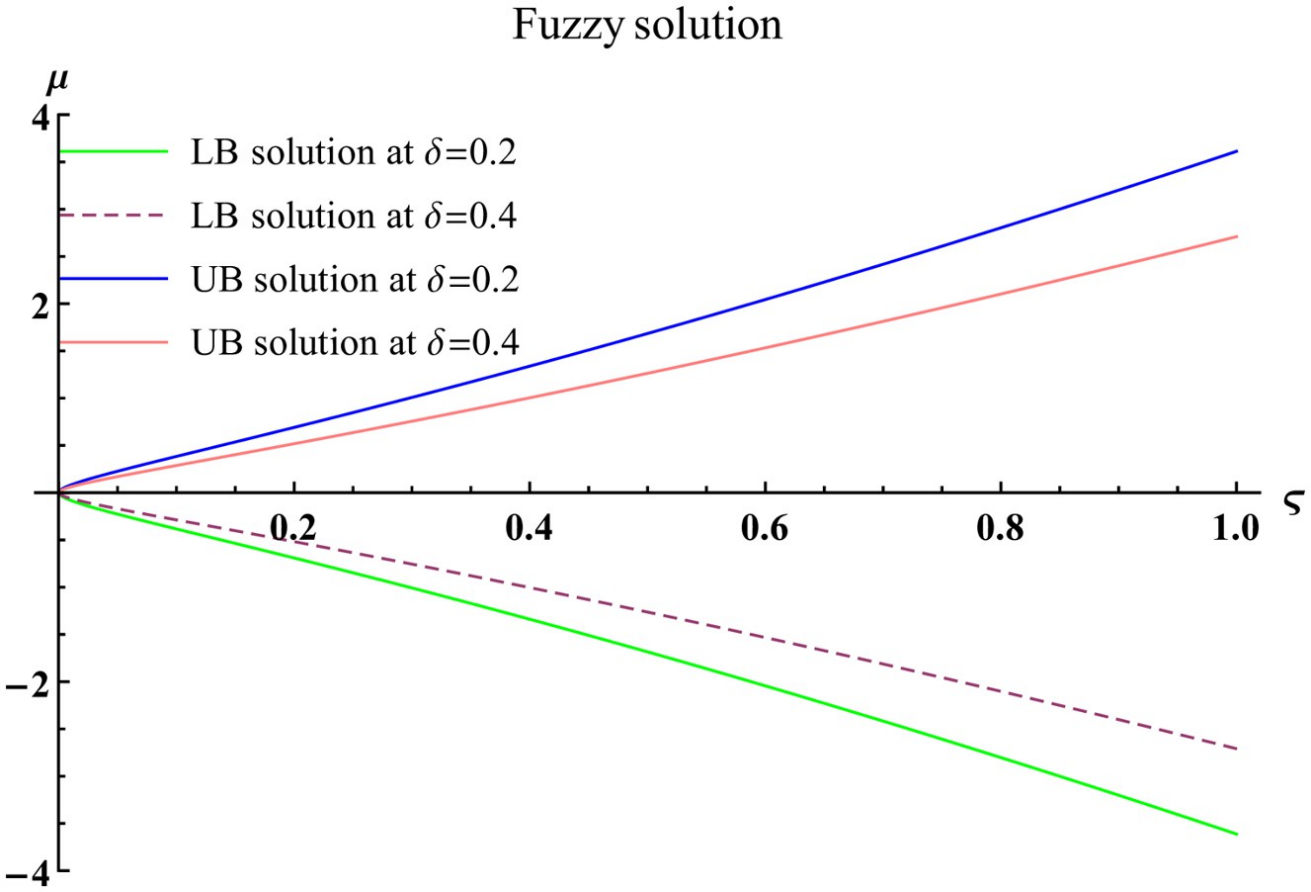
2D fuzzy plot of $\tilde{\vartheta }\left(\varsigma ,\mu ,\tau \right)$ upon *α* = 0.75.

**Fig 10:**
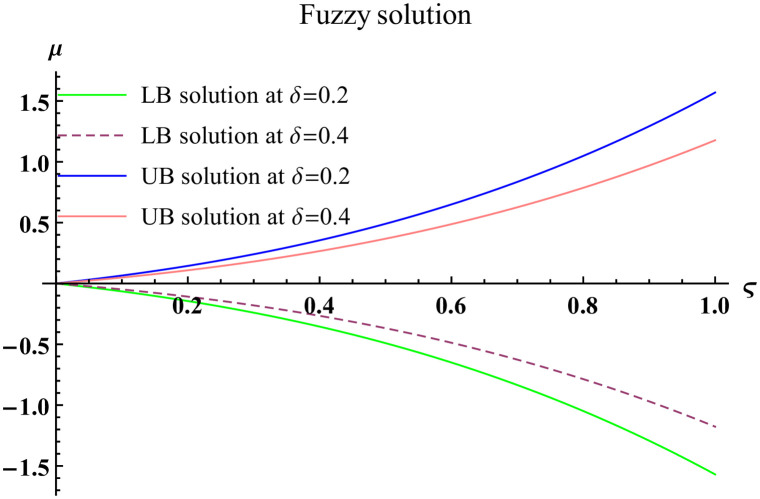
2D fuzzy plot of $\tilde{\vartheta }\left(\varsigma ,\mu ,\tau \right)$ upon *α* = 1.

We explain the physical nature of Example (4.1) in its several graphical structures. We include surface and contour plots that demonstrate the physical nature of the fuzzy heat fractional problem for lower bound approximation. Figs 1 and 2 state the 3D graphical depictions of lower bound approximation at different coordinates of parameters at *τ* = 0.5 with −1 ≤ *ς* ≤ 1 and 0 ≤ *μ* ≤ 1 for *α* = 0.75 and *α* = 1 respectively. Similarly, Figs 3 and 4 show the contour plots of lower bound approximation at different coordinates of parameters at *τ* = 0.5 with −2 ≤ *ς* ≤ 2 and 0 ≤ *μ* ≤ 2 for *α* = 0.75 and *α* = 1 respectively. We include surface and contour plots that demonstrate the physical nature of the fuzzy heat fractional problem for upper bound approximation. Figs 5 and 6 state the 3D graphical depictions of upper bound approximation at different coordinates of parameters at *τ* = 0.5 with 0 ≤ *ς* ≤ 1 and 0 ≤ *μ* ≤ 1 for *α* = 0.75 and *α* = 1 respectively. Similarly, Figs 7 and 8 show the contour plots of upper bound approximation at different coordinates of parameters at *τ* = 0.5 with 0 ≤ *ς* ≤ 2 and 0 ≤ *μ* ≤ 2 for *α* = 0.75 and *α* = 1 respectively. In Figs 9 and 10, we demonstrate a 2D fuzzy structure for lower and upper bound approximation across the parameters *δ* = 0.2 and *δ* = 0.4. Our graphical structures of 3D and 2D show that the suggested scheme is valid and consistent for fuzzy fractional scenarios.

### 4.2 Example 2

Suppose the following 2D fuzzy heat problem of fractional order as follows

$$\begin{array}{c} D_{\tau }^{\alpha }\tilde{\vartheta }\left(\varsigma ,\mu ,\tau \right)={\tilde{\vartheta }}_{\varsigma \varsigma }\left(\varsigma ,\mu ,\tau \right)+{\tilde{\vartheta }}_{\mu \mu }\left(\varsigma ,\mu ,\tau \right)+\varsigma +\mu +\tau^{2}, \end{array}$$ (23)

with following constraints

$$\begin{array}{c} \tilde{\vartheta }\left(\varsigma ,\mu ,0\right)=\tilde{f}\;\text{sin}\;\left[\pi \left(\varsigma +\mu \right)\right]. \end{array}$$ (24)

here $\tilde{f}=\left[\underline{\omega },\bar{\omega }\right]=\left[\delta -1,1-\delta \right]$.

#### 4.2.1 Lower bound findings for 2D fuzzy problem

The lower bound fuzzy problem can be derived from Eq (23) as follows

$$\begin{array}{c} \frac{\partial^{\alpha }\underline{\vartheta }}{\partial \tau^{\alpha }}=\frac{\partial^{2}\underline{\vartheta }}{\partial \varsigma^{2}}+\frac{\partial^{2}\underline{\vartheta }}{\partial \mu^{2}}+\varsigma +\mu +\tau^{2}, \end{array}$$ (25)

with lower fuzzy initial constraints

$$\begin{array}{c} \underline{\vartheta_{0}}\left(\varsigma ,\mu ,\tau \right)=\left(\delta -1\right)\;\text{sin}\;\left[\pi \left(\varsigma +\mu \right)\right]. \end{array}$$ (26)

By employing the SRPSS, we obtain the following results:

$$\begin{array}{cc} \underline{\vartheta_{1}}\left(\varsigma ,\mu ,\tau \right) & =-2\left(\delta -1\right)\pi^{2}\;\text{sin}\;\left[\pi \left(\varsigma +\mu \right)\right]\frac{\tau^{\alpha }}{\Gamma (\alpha +1)},\\ \underline{\vartheta_{2}}\left(\varsigma ,\mu ,\tau \right) & =4\left(\delta -1\right)\pi^{2}\;\text{sin}\;\left[\pi \left(\varsigma +\mu \right)\right]\frac{\tau^{2\alpha }}{\Gamma (2\alpha +1)},\\ \underline{\vartheta_{3}}\left(\varsigma ,\mu ,\tau \right) & =-8\left(\delta -1\right)\pi^{2}\;\text{sin}\;\left[\pi \left(\varsigma +\mu \right)\right]\frac{\tau^{3\alpha }}{\Gamma (3\alpha +1)},\\ & \vdots . \end{array}$$

Therefore, we may organize it into the appropriate sequence

$$\begin{array}{c} \begin{array}{cc} \underline{\vartheta }\left(\varsigma ,\mu ,\tau \right) & =\underline{\vartheta_{0}}\left(\varsigma ,\mu ,\tau \right)+\underline{\vartheta_{1}}\left(\varsigma ,\mu ,\tau \right)+\underline{\vartheta_{2}}\left(\varsigma ,\mu ,\tau \right)+\underline{\vartheta_{3}}\left(\varsigma ,\mu ,\tau \right)+\cdots ,\\ & =\left(\delta -1\right)\;\text{sin}\;\left[\pi \left(\varsigma +\mu \right)\right]+\varsigma \frac{\tau^{\alpha }}{\Gamma (\alpha +1)}+\delta \frac{\tau^{\alpha }}{\Gamma (\alpha +1)}+2\frac{\tau^{\alpha +2}}{\Gamma (\alpha +2)}\\ & -2\left(\delta -1\right)\pi^{2}\;\text{sin}\;\left[\pi \left(\varsigma +\mu \right)\right]\frac{\tau^{\alpha }}{\Gamma (\alpha +1)}+4\left(\delta -1\right)\pi^{2}\;\text{sin}\;\left[\pi \left(\varsigma +\mu \right)\right]\frac{\tau^{2\alpha }}{\Gamma (2\alpha +1)}\\ & -8\left(\delta -1\right)\pi^{2}\;\text{sin}\;\left[\pi \left(\varsigma +\mu \right)\right]\frac{\tau^{3\alpha }}{\Gamma (3\alpha +1)}+\cdots . \end{array} \end{array}$$ (27)

**Note:** When $\tilde{g}\left(\varsigma ,\mu ,\tau \right)=0$, the preceding equation is transformed as

$$\begin{array}{c} \begin{array}{cc} \underline{\vartheta }\left(\varsigma ,\mu ,\tau \right) & =\left(\delta -1\right)\;\text{sin}\;\left[\pi \left(\varsigma +\mu \right)\right]-2\left(\delta -1\right)\pi^{2}\;\text{sin}\;\left[\pi \left(\varsigma +\mu \right)\right]\frac{\tau^{\alpha }}{\Gamma (\alpha +1)}\\ & +4\left(\delta -1\right)\pi^{2}\;\text{sin}\;\left[\pi \left(\varsigma +\mu \right)\right]\frac{\tau^{2\alpha }}{\Gamma (2\alpha +1)}-8\left(\delta -1\right)\pi^{2}\;\text{sin}\;\left[\pi \left(\varsigma +\mu \right)\right]\frac{\tau^{3\alpha }}{\Gamma (3\alpha +1)}+\cdots . \end{array} \end{array}$$ (28)

This series turns to closed form solutions such as

$$\begin{array}{c} \underline{\vartheta }\left(\varsigma ,\mu ,\tau \right)=\left(\delta -1\right)\;\text{sin}\;\left[\pi \left(\varsigma +\mu \right)\right]\sum_{n=0}^{\infty }\frac{{\left(-1\right)}^{n}{\left(2\pi^{2}\tau^{\alpha }\right)}^{n}}{\Gamma (n\alpha +1)}. \end{array}$$ (29)

#### 4.2.2 Upper bound findings for 2D fuzzy problem

The upper bound fuzzy problem can be derived from Eq (23) as follows

$$\begin{array}{c} \frac{\partial^{\alpha }\bar{\vartheta }}{\partial \tau^{\alpha }}=\frac{\partial^{2}\bar{\vartheta }}{\partial \varsigma^{2}}+\frac{\partial^{2}\bar{\vartheta }}{\partial \mu^{2}}+\varsigma +\mu +\tau^{2}, \end{array}$$ (30)

with upper fuzzy initial constraints

$$\begin{array}{c} \bar{\vartheta_{0}}\left(\varsigma ,\mu ,\tau \right)=\left(1-\delta \right)\;\text{sin}\;\left[\pi \left(\varsigma +\mu \right)\right]. \end{array}$$ (31)

By employing the SRPSS, we obtain the following results:

$$\begin{array}{cc} \bar{\vartheta_{1}}\left(\varsigma ,\mu ,\tau \right) & =-2\left(1-\delta \right)\pi^{2}\;\text{sin}\;\left[\pi \left(\varsigma +\mu \right)\right]\frac{\tau^{\alpha }}{\Gamma (\alpha +1)},\\ \bar{\vartheta_{2}}\left(\varsigma ,\mu ,\tau \right) & =4\left(1-\delta \right)\pi^{2}\;\text{sin}\;\left[\pi \left(\varsigma +\mu \right)\right]\frac{\tau^{2\alpha }}{\Gamma (2\alpha +1)},\\ \bar{\vartheta_{3}}\left(\varsigma ,\mu ,\tau \right) & =-8\left(1-\delta \right)\pi^{2}\;\text{sin}\;\left[\pi \left(\varsigma +\mu \right)\right]\frac{\tau^{3\alpha }}{\Gamma (3\alpha +1)},\\ & \vdots . \end{array}$$

Therefore, we may organize it into the appropriate sequence

$$\begin{array}{c} \begin{array}{cc} \bar{\vartheta }\left(\varsigma ,\mu ,\tau \right) & =\bar{\vartheta_{0}}\left(\varsigma ,\mu ,\tau \right)+\bar{\vartheta_{1}}\left(\varsigma ,\mu ,\tau \right)+\bar{\vartheta_{2}}\left(\varsigma ,\mu ,\tau \right)+\bar{\vartheta_{3}}\left(\varsigma ,\mu ,\tau \right)+\cdots ,\\ & =\left(1-\delta \right)\;\text{sin}\;\left[\pi \left(\varsigma +\mu \right)\right]+\varsigma \frac{\tau^{\alpha }}{\Gamma (\alpha +1)}+\mu \frac{\tau^{\alpha }}{\Gamma (\alpha +1)}+2\frac{\tau^{\alpha +2}}{\Gamma (\alpha +2)}\\ & -2\left(1-\delta \right)\pi^{2}\;\text{sin}\;\left[\pi \left(\varsigma +\mu \right)\right]\frac{\tau^{\alpha }}{\Gamma (\alpha +1)}+4\left(1-\delta \right)\pi^{2}\;\text{sin}\;\left[\pi \left(\varsigma +\mu \right)\right]\frac{\tau^{2\alpha }}{\Gamma (2\alpha +1)}\\ & -8\left(1-\delta \right)\pi^{2}\;\text{sin}\;\left[\pi \left(\varsigma +\mu \right)\right]\frac{\tau^{3\alpha }}{\Gamma (3\alpha +1)}+\cdots . \end{array} \end{array}$$ (32)

**Note:** When $\tilde{g}\left(\varsigma ,\mu ,\tau \right)=0$, the preceding equation is transformed into

$$\begin{array}{c} \begin{array}{cc} \bar{\vartheta }\left(\varsigma ,\mu ,\tau \right) & =\left(1-\delta \right)\;\text{sin}\;\left[\pi \left(\varsigma +\mu \right)\right]-2\left(1-\delta \right)\pi^{2}\;\text{sin}\;\left[\pi \left(\varsigma +\mu \right)\right]\frac{\tau^{\alpha }}{\Gamma (\alpha +1)}\\ & +4\left(1-\delta \right)\pi^{2}\;\text{sin}\;\left[\pi \left(\varsigma +\mu \right)\right]\frac{\tau^{2\alpha }}{\Gamma (2\alpha +1)}-8\left(1-\delta \right)\pi^{2}\;\text{sin}\;\left[\pi \left(\varsigma +\mu \right)\right]\frac{\tau^{3\alpha }}{\Gamma (3\alpha +1)}+\cdots . \end{array} \end{array}$$ (33)

This series turns to closed form solutions such as

$$\begin{array}{c} \bar{\vartheta }\left(\varsigma ,\mu ,\tau \right)=\left(1-\delta \right)\;\text{sin}\;\left[\pi \left(\varsigma +\mu \right)\right]\sum_{n=0}^{\infty }\frac{{\left(-1\right)}^{n}{\left(2\pi^{2}\tau^{\alpha }\right)}^{n}}{\Gamma (n\alpha +1)}. \end{array}$$ (34)

**Fig 11:**
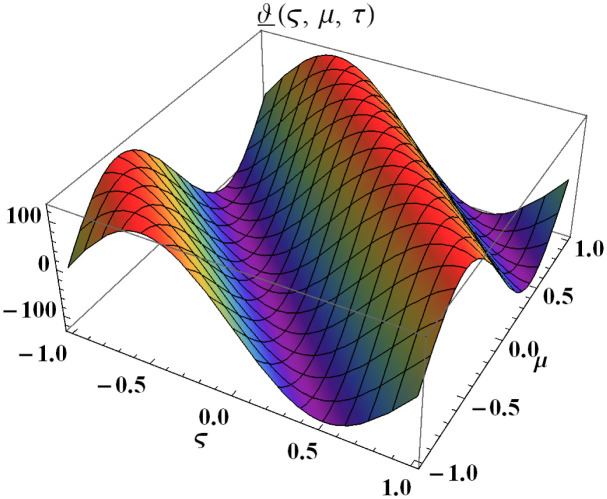
Surface structure of $\underline{\vartheta }\left(\varsigma ,\mu ,\tau \right)$ upon *α* = 0.75.

**Fig 12:**
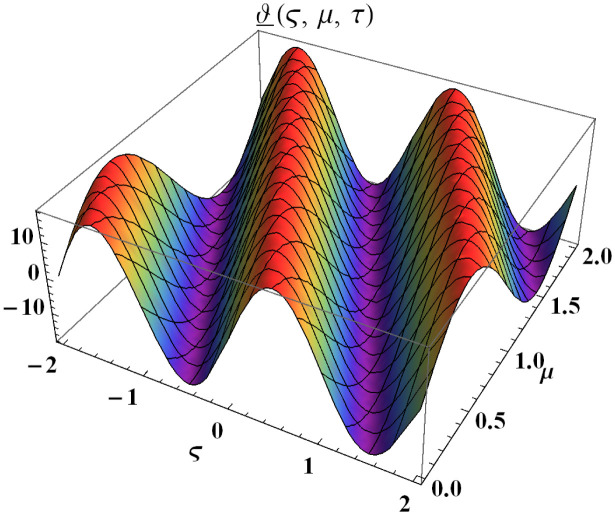
Surface structure of $\underline{\vartheta }\left(\varsigma ,\mu ,\tau \right)$ upon *α* = 1.

**Fig 13:**
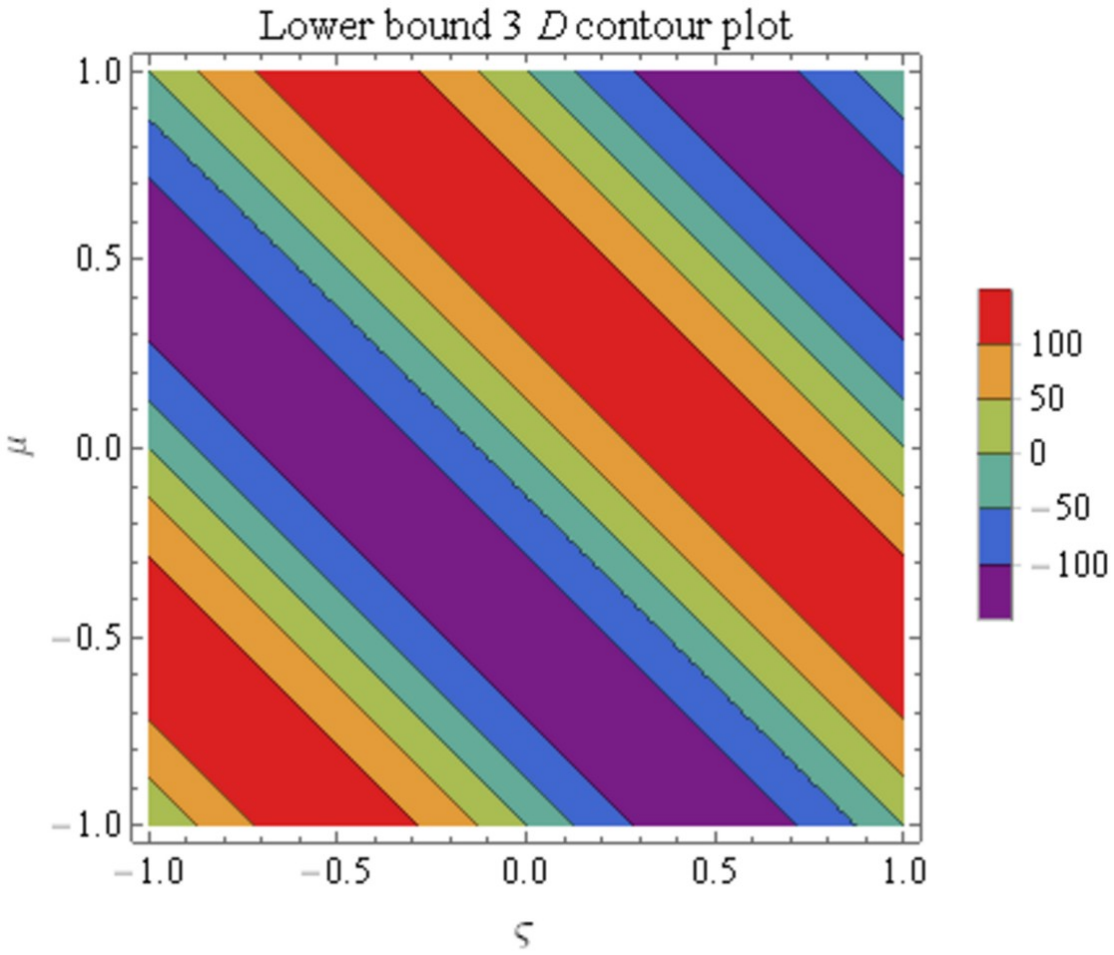
Contour structure of $\underline{\vartheta }\left(\varsigma ,\mu ,\tau \right)$ upon *α* = 0.75.

**Fig 14:**
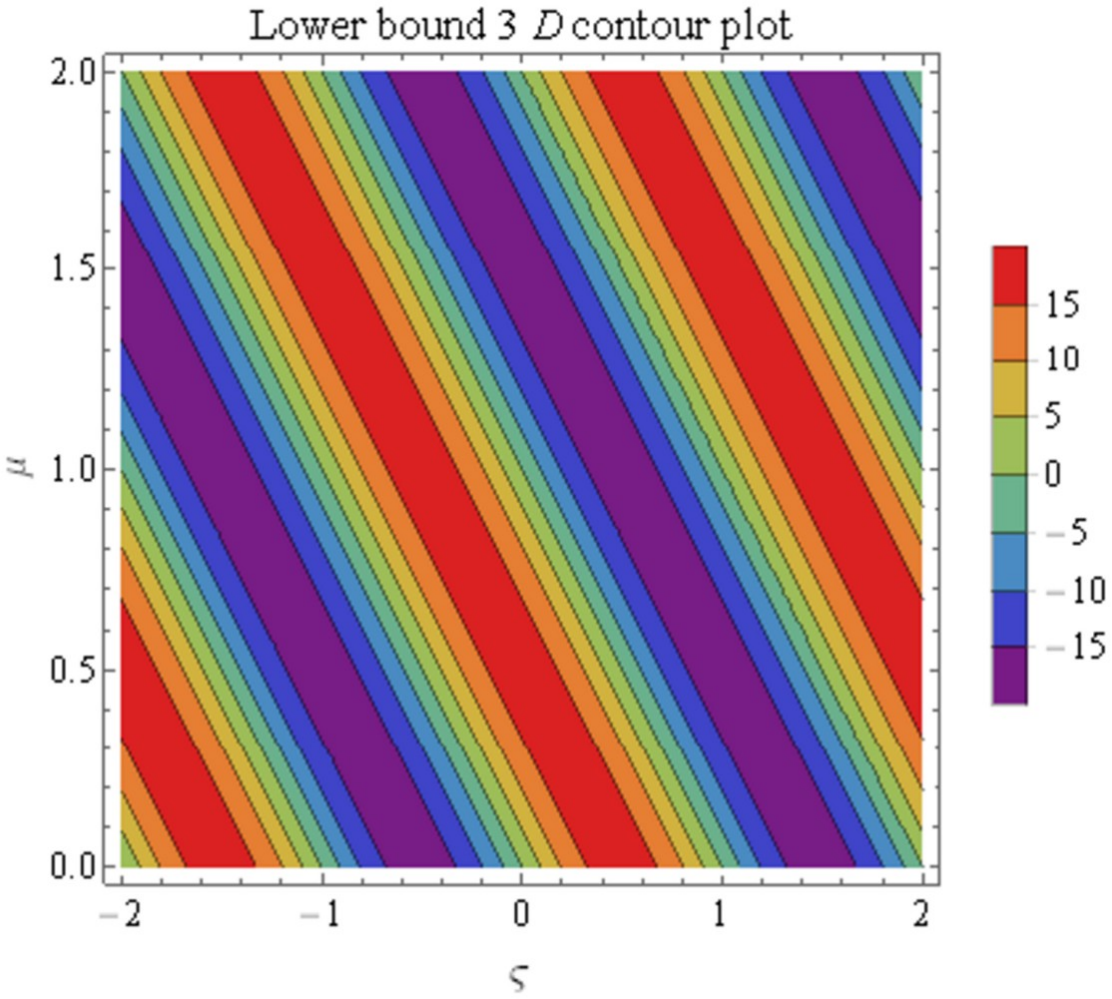
Contour structure of $\underline{\vartheta }\left(\varsigma ,\mu ,\tau \right)$ upon *α* = 1.

**Fig 15:**
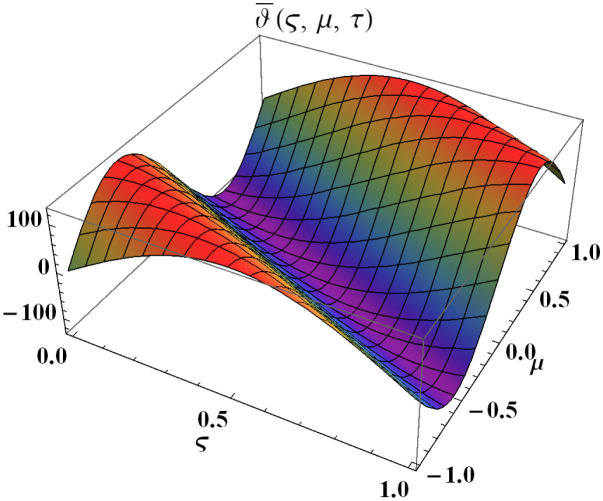
Surface structure of $\bar{\vartheta }\left(\varsigma ,\mu ,\tau \right)$ upon *α* = 0.75.

**Fig 16:**
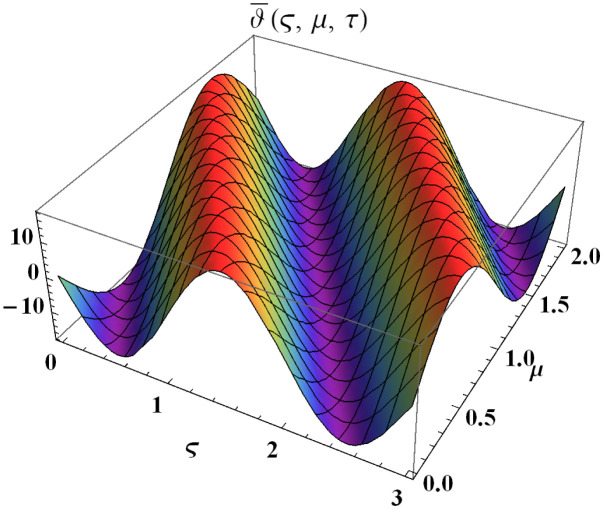
Surface structure of $\bar{\vartheta }\left(\varsigma ,\mu ,\tau \right)$ upon *α* = 1.

**Fig 17:**
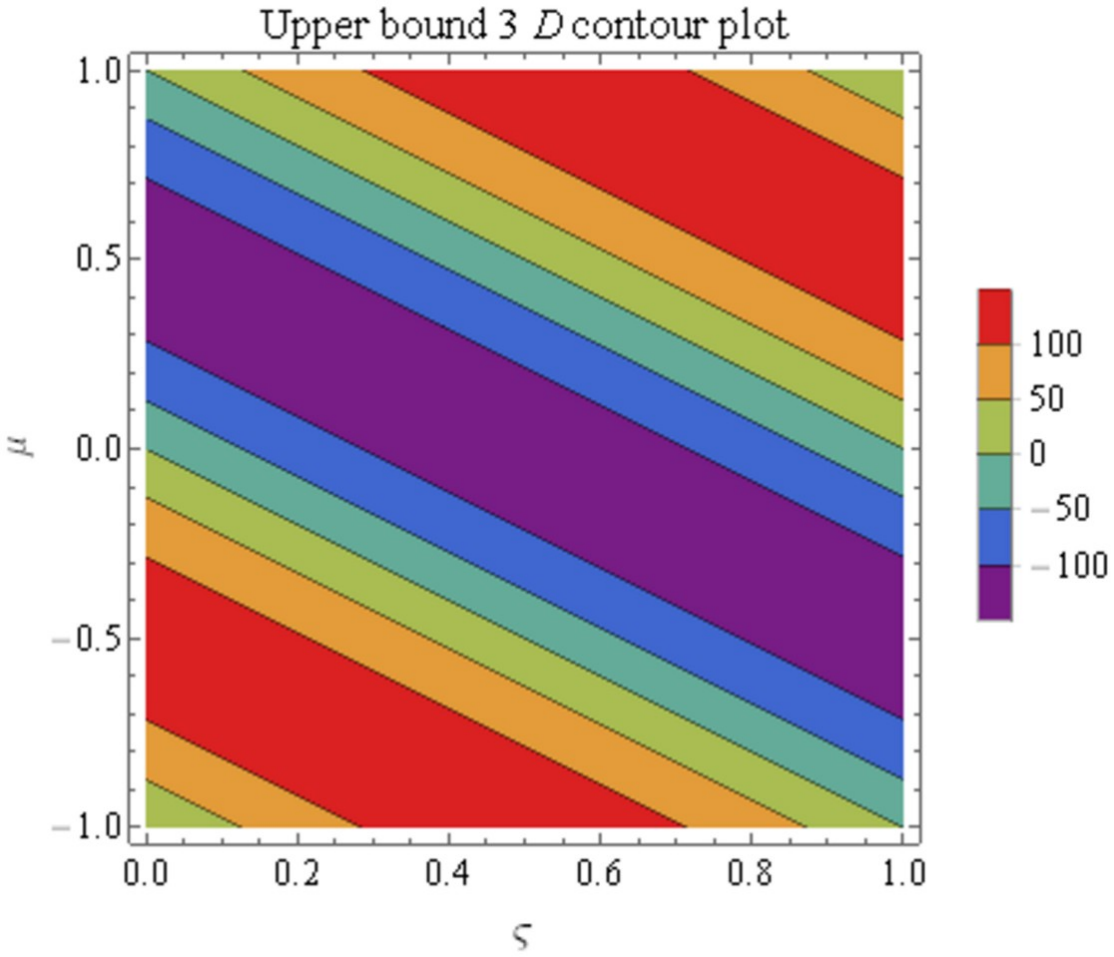
Contour structure of $\bar{\vartheta }\left(\varsigma ,\mu ,\tau \right)$ upon *α* = 0.75.

**Fig 18:**
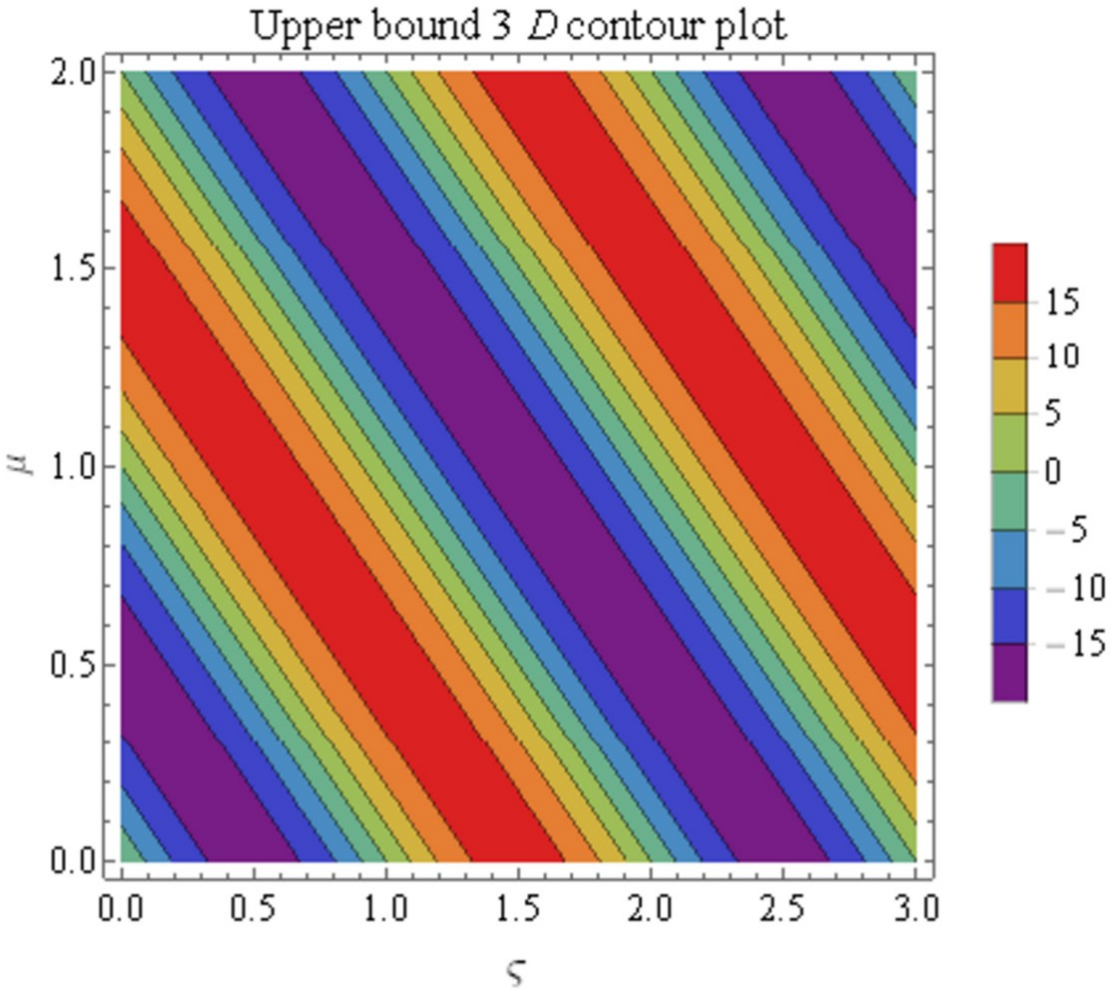
Contour structure of $\bar{\vartheta }\left(\varsigma ,\mu ,\tau \right)$ upon *α* = 1.

**Fig 19:**
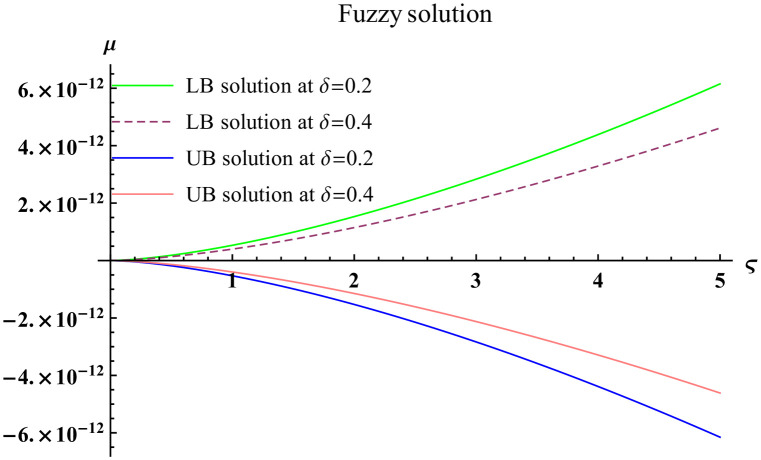
2D fuzzy plot of $\tilde{\vartheta }\left(\varsigma ,\mu ,\tau \right)$ upon *α* = 0.75.

**Fig 20:**
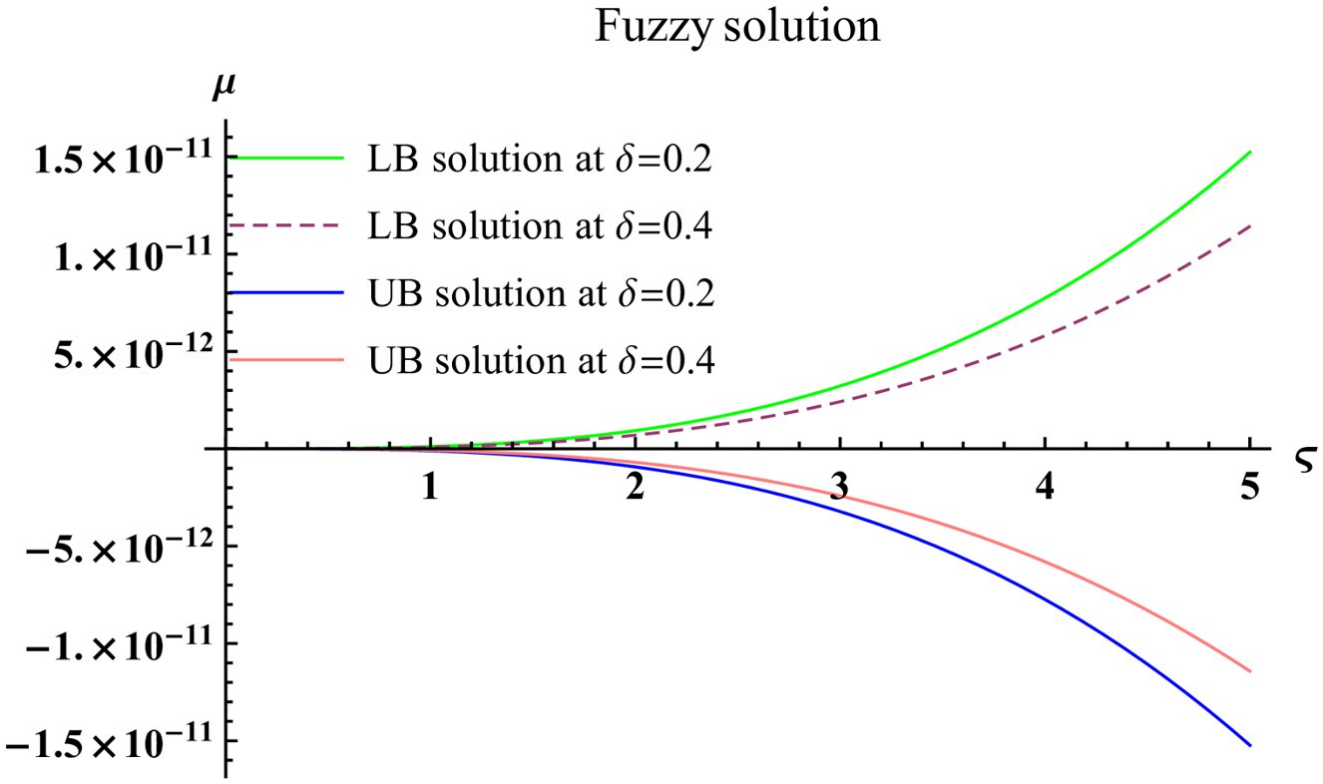
2D fuzzy plot of $\tilde{\vartheta }\left(\varsigma ,\mu ,\tau \right)$ upon *α* = 1.

We explain the physical nature of Example (4.2) in its several graphical structures. We include surface and contour plots that demonstrate the physical nature of the fuzzy heat fractional problem for lower bound approximation. Figs 11 and 12 state the 3D graphical depictions of lower bound approximation at different coordinates of parameters at *τ* = 0.3 with −1 ≤ *ς* ≤ 1 and −1 ≤ *μ* ≤ 1 for *α* = 0.75 and *α* = 1 respectively. Similarly, Figs 13 and 14 show the contour plots of lower bound approximation at different coordinates of parameters at *τ* = 0.3 with −2 ≤ *ς* ≤ 2 and 0 ≤ *μ* ≤ 2 for *α* = 0.75 and *α* = 1 respectively. We include surface and contour plots that demonstrate the physical nature of the fuzzy heat fractional problem for upper bound approximation. Figs 15 and 16 state the 3D graphical depictions of upper bound approximation at different coordinates of parameters at *τ* = 0.3 with 0 ≤ *ς* ≤ 1 and −1 ≤ *μ* ≤ 1 for *α* = 0.75 and *α* = 1 respectively. Similarly, Figs 17 and 18 show the contour plots of upper bound approximation at different coordinates of parameters at *τ* = 0.3 with 0 ≤ *ς* ≤ 3 and 0 ≤ *μ* ≤ 2 for *α* = 0.75 and *α* = 1 respectively. In Figs 19 and 20, we demonstrate a 2D fuzzy structure for lower and upper bound approximation across the parameters *δ* = 0.2 and *δ* = 0.4. Our graphical structures of 3D and 2D show that the suggested scheme is valid and consistent for fuzzy fractional scenarios.

### 4.3 Example 3

Suppose the following 2D fuzzy heat problem of fractional order as follows

$$\begin{array}{c} D_{\tau }^{\alpha }\tilde{\vartheta }\left(\varsigma ,\mu ,\tau \right)=\frac{1}{2}{\left(\varsigma +\mu \right)}^{2}[{\tilde{\vartheta }}_{\varsigma \varsigma }\left(\varsigma ,\mu ,\tau \right)+{\tilde{\vartheta }}_{\mu \mu }\left(\varsigma ,\mu ,\tau \right)]+\varsigma +\mu +\tau^{4}, \end{array}$$ (35)

with following constraints

$$\begin{array}{c} \tilde{\vartheta }\left(\varsigma ,\mu ,0\right)=\tilde{f}{\left(\varsigma +\mu \right)}^{2}. \end{array}$$ (36)

with $\tilde{f}=\left[\underline{f},\bar{f}\right]=\left[\delta -1,1-\delta \right]$.

#### 4.3.1 Lower bound findings for 2D fuzzy problem

The lower bound fuzzy problem can be derived from Eq (35) as follows

$$\begin{array}{c} \frac{\partial^{\alpha }\underline{\vartheta }}{\partial \tau^{\alpha }}=\frac{1}{2}{\left(\varsigma +\mu \right)}^{2}\frac{\partial^{2}\underline{\vartheta }}{\partial \varsigma^{2}}+\frac{\partial^{2}\underline{\vartheta }}{\partial \mu^{2}}+\varsigma +\mu +\tau^{4}, \end{array}$$ (37)

with lower fuzzy initial constraints

$$\begin{array}{c} \underline{\vartheta_{0}}\left(\varsigma ,\mu ,\tau \right)=\left(\delta -1\right){\left(\varsigma +\mu \right)}^{2}. \end{array}$$ (38)

By employing the SRPSS, one can compute the following results:

$$\begin{array}{cc} \underline{\vartheta_{1}}\left(\varsigma ,\mu ,\tau \right) & =2\left(\delta -1\right){\left(\varsigma +\mu \right)}^{2}\frac{\tau^{\alpha }}{\Gamma (\alpha +1)},\\ \underline{\vartheta_{2}}\left(\varsigma ,\mu ,\tau \right) & =4\left(\delta -1\right){\left(\varsigma +\mu \right)}^{2}\frac{\tau^{2\alpha }}{\Gamma (2\alpha +1)},\\ \underline{\vartheta_{3}}\left(\varsigma ,\mu ,\tau \right) & =8\left(\delta -1\right){\left(\varsigma +\mu \right)}^{2}\frac{\tau^{3\alpha }}{\Gamma (3\alpha +1)},\\ & \vdots . \end{array}$$

Therefore, we may organize it into the appropriate sequence

$$\begin{array}{c} \begin{array}{cc} \underline{\vartheta }\left(\varsigma ,\mu ,\tau \right) & =\left(\delta -1\right){\left(\varsigma +\mu \right)}^{2}+\varsigma \frac{\tau^{\alpha }}{\Gamma (\alpha +1)}+\mu \frac{\tau^{\alpha }}{\Gamma (\alpha +1)}+24\frac{\tau^{\alpha +4}}{\Gamma (\alpha +5)}+2\left(\delta -1\right){\left(\varsigma +\mu \right)}^{2}\frac{\tau^{\alpha }}{\Gamma (\alpha +1)}\\ & +4\left(\delta -1\right){\left(\varsigma +\mu \right)}^{2}\frac{\tau^{2\alpha }}{\Gamma (2\alpha +1)}+8\left(\delta -1\right){\left(\varsigma +\mu \right)}^{2}\frac{\tau^{3\alpha }}{\Gamma (3\alpha +1)}+\cdots . \end{array} \end{array}$$ (39)

**Note:** When $\tilde{g}\left(\varsigma ,\mu ,\tau \right)=0$, the preceding equation is transformed as

$$\begin{array}{c} \begin{array}{cc} \underline{\vartheta }\left(\varsigma ,\mu ,\tau \right) & =\left(\delta -1\right){\left(\varsigma +\mu \right)}^{2}+2\left(\delta -1\right){\left(\varsigma +\mu \right)}^{2}\frac{\tau^{\alpha }}{\Gamma (\alpha +1)}\\ & +4\left(\delta -1\right){\left(\varsigma +\mu \right)}^{2}\frac{\tau^{2\alpha }}{\Gamma (2\alpha +1)}+8\left(\delta -1\right){\left(\varsigma +\mu \right)}^{2}\frac{\tau^{3\alpha }}{\Gamma (3\alpha +1)}+\cdots . \end{array} \end{array}$$ (40)

This series turns to closed form solutions such as

$$\begin{array}{c} \underline{\vartheta }\left(\varsigma ,\mu ,\tau \right)=\left(\delta -1\right){\left(\varsigma +\mu \right)}^{2}\;\sum_{n=0}^{\infty }\frac{{\left(2\tau^{\alpha }\right)}^{n}}{\Gamma (n\alpha +1)}. \end{array}$$ (41)

#### 4.3.2 Upper bound findings for 2D fuzzy problem

The upper bound fuzzy problem can be derived from Eq (35) as follows

$$\begin{array}{c} \frac{\partial^{\alpha }\bar{\vartheta }}{\partial \tau^{\alpha }}=\frac{1}{2}{\left(\varsigma +\mu \right)}^{2}\frac{\partial^{2}\bar{\vartheta }}{\partial \varsigma^{2}}+\frac{\partial^{2}\bar{\vartheta }}{\partial \mu^{2}}+\varsigma +\mu +\tau^{4}, \end{array}$$ (42)

with upper fuzzy initial constraints

$$\begin{array}{c} \bar{\vartheta_{0}}\left(\varsigma ,\mu ,\tau \right)=\left(1-\delta \right){\left(\varsigma +\mu \right)}^{2}. \end{array}$$ (43)

By employing the SRPSS, we obtain the following results:

$$\begin{array}{cc} \bar{\vartheta_{1}}\left(\varsigma ,\mu ,\tau \right) & =2\left(1-\delta \right){\left(\varsigma +\mu \right)}^{2}\frac{\tau^{\alpha }}{\Gamma (\alpha +1)},\\ \bar{\vartheta_{2}}\left(\varsigma ,\mu ,\tau \right) & =4\left(1-\delta \right){\left(\varsigma +\mu \right)}^{2}\frac{\tau^{2\alpha }}{\Gamma (2\alpha +1)},\\ \bar{\vartheta_{3}}\left(\varsigma ,\mu ,\tau \right) & =8\left(1-\delta \right){\left(\varsigma +\mu \right)}^{2}\frac{\tau^{3\alpha }}{\Gamma (3\alpha +1)},\\ & \vdots . \end{array}$$

Therefore, we may organize it into the appropriate sequence

$$\begin{array}{c} \begin{array}{cc} \bar{\vartheta }\left(\varsigma ,\mu ,\tau \right) & =\left(1-\delta \right){\left(\varsigma +\mu \right)}^{2}+\varsigma \frac{\tau^{\alpha }}{\Gamma (\alpha +1)}+\mu \frac{\tau^{\alpha }}{\Gamma (\alpha +1)}+24\frac{\tau^{\alpha +4}}{\Gamma (\alpha +5)}+2\left(1-\delta \right){\left(\varsigma +\mu \right)}^{2}\frac{\tau^{\alpha }}{\Gamma (\alpha +1)}\\ & +4\left(1-\delta \right){\left(\varsigma +\mu \right)}^{2}\frac{\tau^{2\alpha }}{\Gamma (2\alpha +1)}+8\left(1-\delta \right){\left(\varsigma +\mu \right)}^{2}\frac{\tau^{3\alpha }}{\Gamma (3\alpha +1)}+\cdots . \end{array} \end{array}$$ (44)

**Note:** When *g*(*ς*, *μ*, *τ*) = 0, the preceding equation is transformed into

$$\begin{array}{c} \begin{array}{cc} \bar{\vartheta }\left(\varsigma ,\mu ,\tau \right) & =\left(1-\delta \right){\left(\varsigma +\mu \right)}^{2}+2\left(1-\delta \right){\left(\varsigma +\mu \right)}^{2}\frac{\tau^{\alpha }}{\Gamma (\alpha +1)}\\ & +4\left(1-\delta \right){\left(\varsigma +\mu \right)}^{2}\frac{\tau^{2\alpha }}{\Gamma (2\alpha +1)}+8\left(1-\delta \right){\left(\varsigma +\mu \right)}^{2}\frac{\tau^{3\alpha }}{\Gamma (3\alpha +1)}+\cdots . \end{array} \end{array}$$ (45)

This series turns to closed form solutions such as

$$\begin{array}{c} \bar{\vartheta }\left(\varsigma ,\mu ,\tau \right)=\left(1-\delta \right){\left(\varsigma +\mu \right)}^{2}\;\sum_{n=0}^{\infty }\frac{{\left(2\tau^{\alpha }\right)}^{n}}{\Gamma (n\alpha +1)}. \end{array}$$ (46)

**Fig 21:**
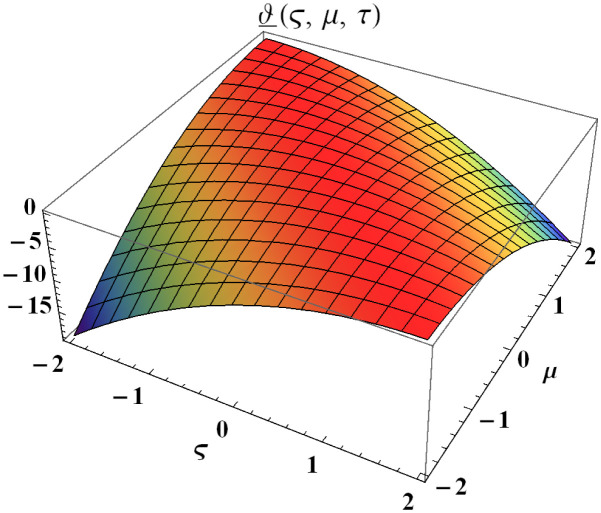
Surface structure of $\underline{\vartheta }\left(\varsigma ,\mu ,\tau \right)$ upon *α* = 0.75.

**Fig 22:**
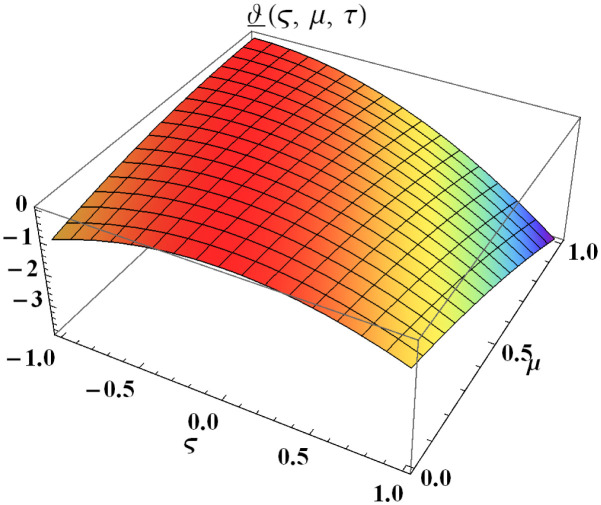
Surface structure of $\underline{\vartheta }\left(\varsigma ,\mu ,\tau \right)$ upon *α* = 1.

**Fig 23:**
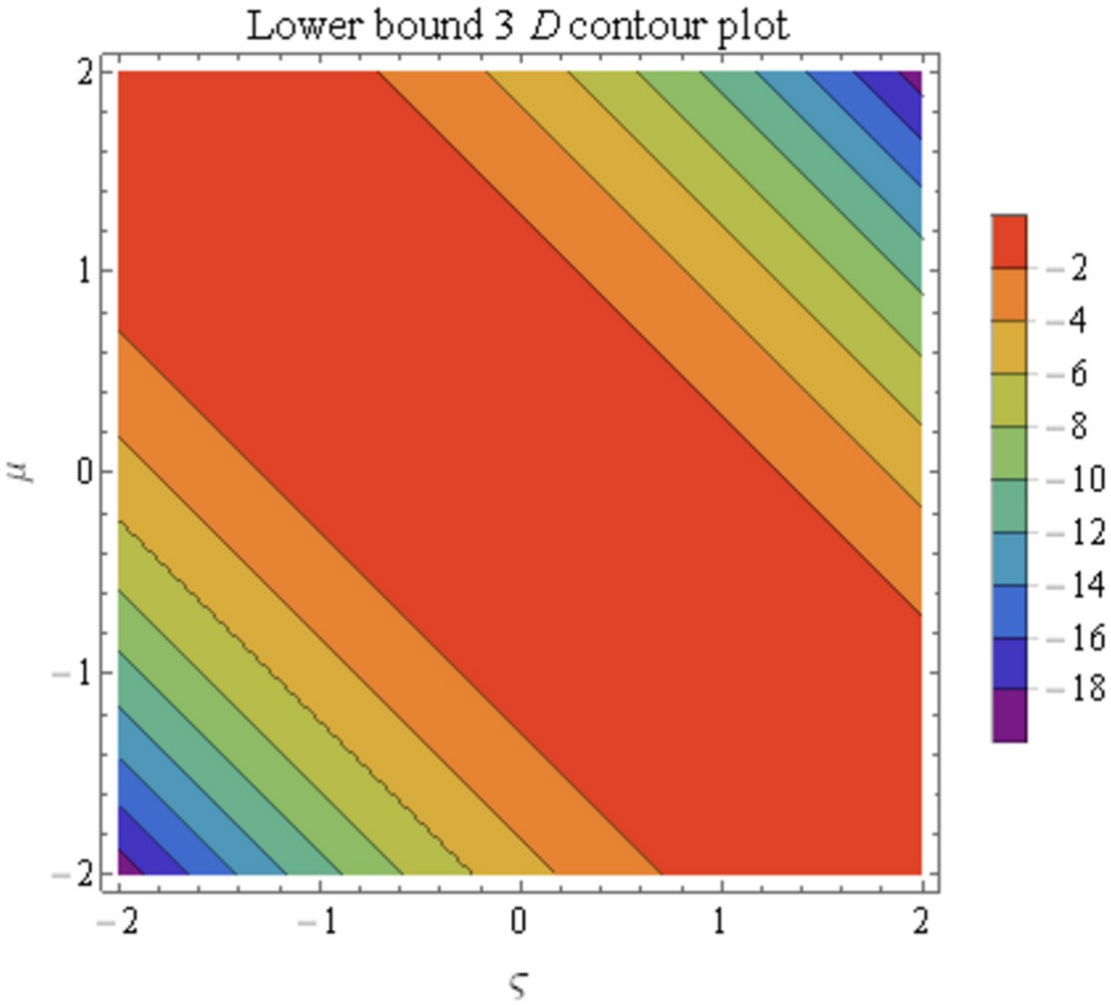
Contour structure of $\underline{\vartheta }\left(\varsigma ,\mu ,\tau \right)$ upon *α* = 0.75.

**Fig 24:**
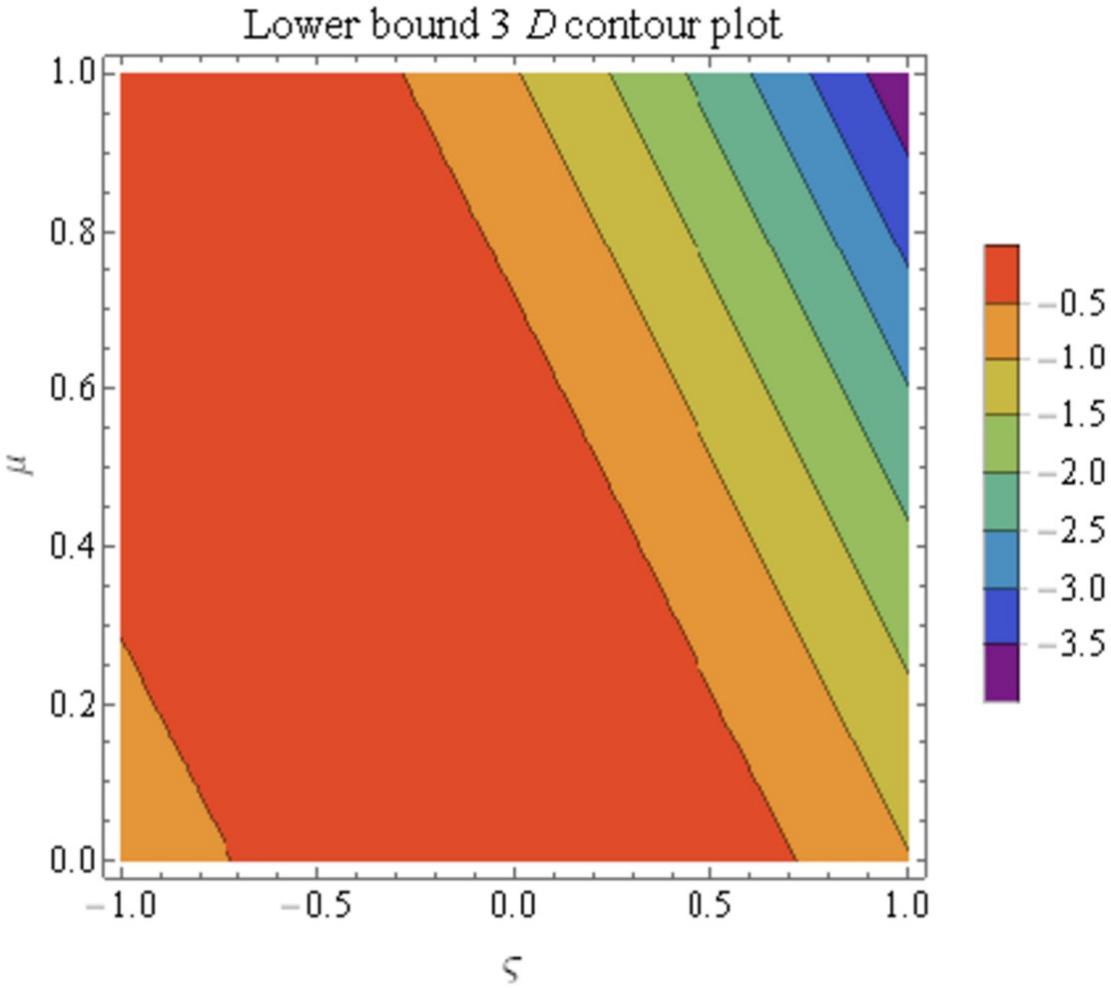
Contour structure of $\underline{\vartheta }\left(\varsigma ,\mu ,\tau \right)$ upon *α* = 1.

**Fig 25:**
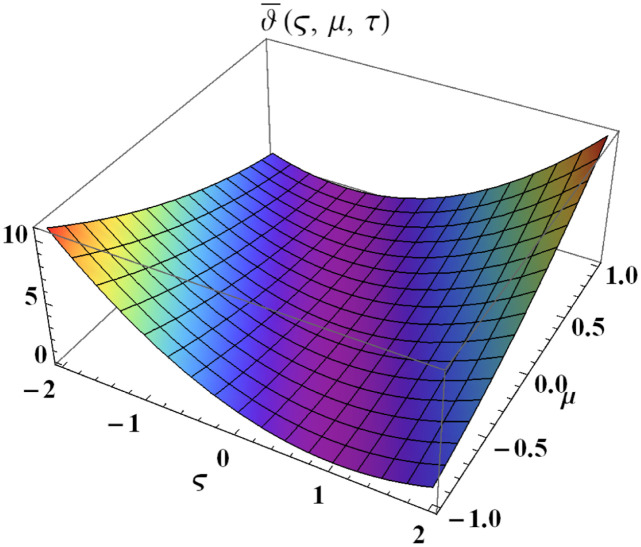
Surface structure of $\bar{\vartheta }\left(\varsigma ,\mu ,\tau \right)$ upon *α* = 0.75.

**Fig 26:**
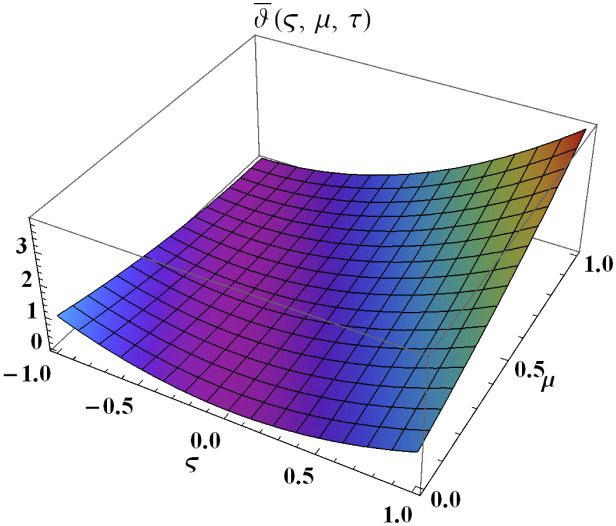
Surface structure of $\bar{\vartheta }\left(\varsigma ,\mu ,\tau \right)$ upon *α* = 1.

**Fig 27:**
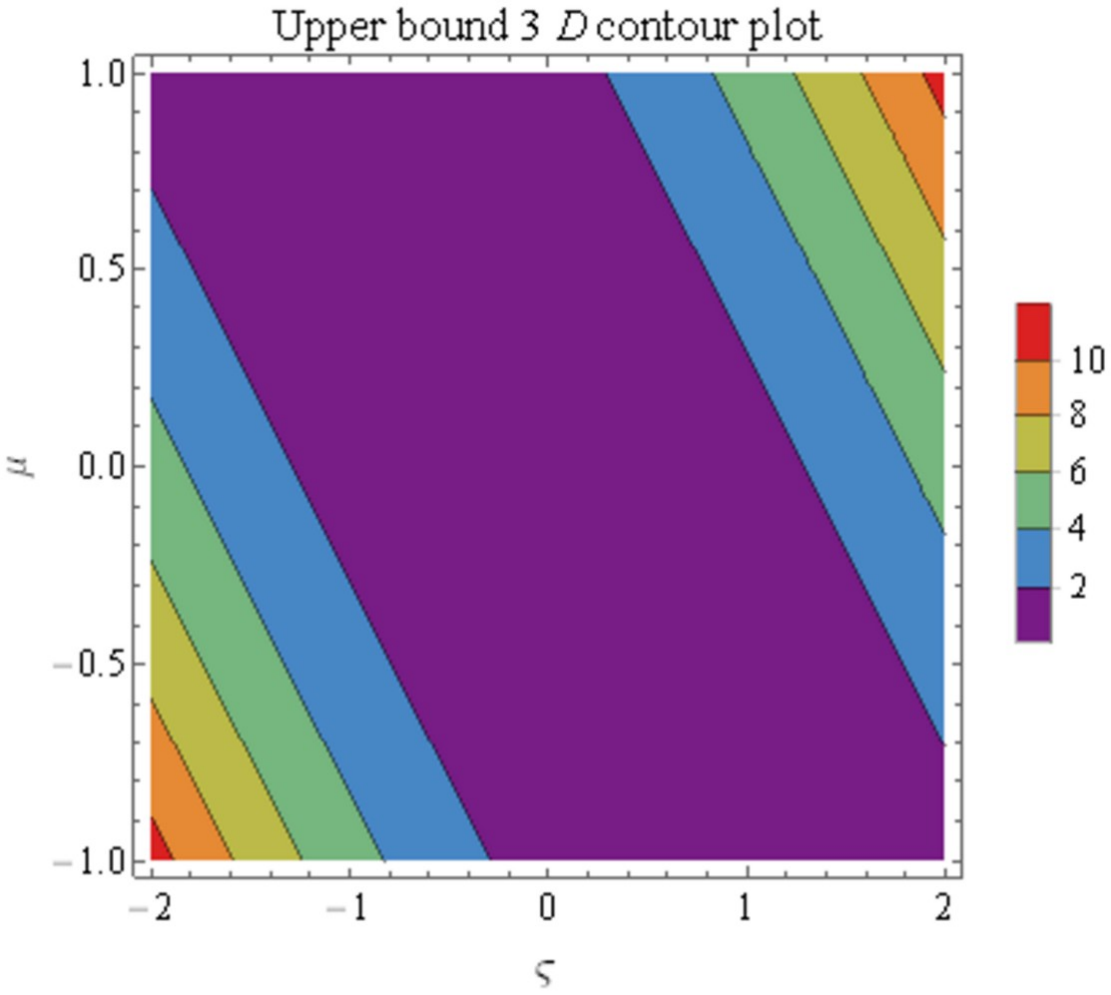
Contour structure of $\bar{\vartheta }\left(\varsigma ,\mu ,\tau \right)$ upon *α* = 0.75.

**Fig 28:**
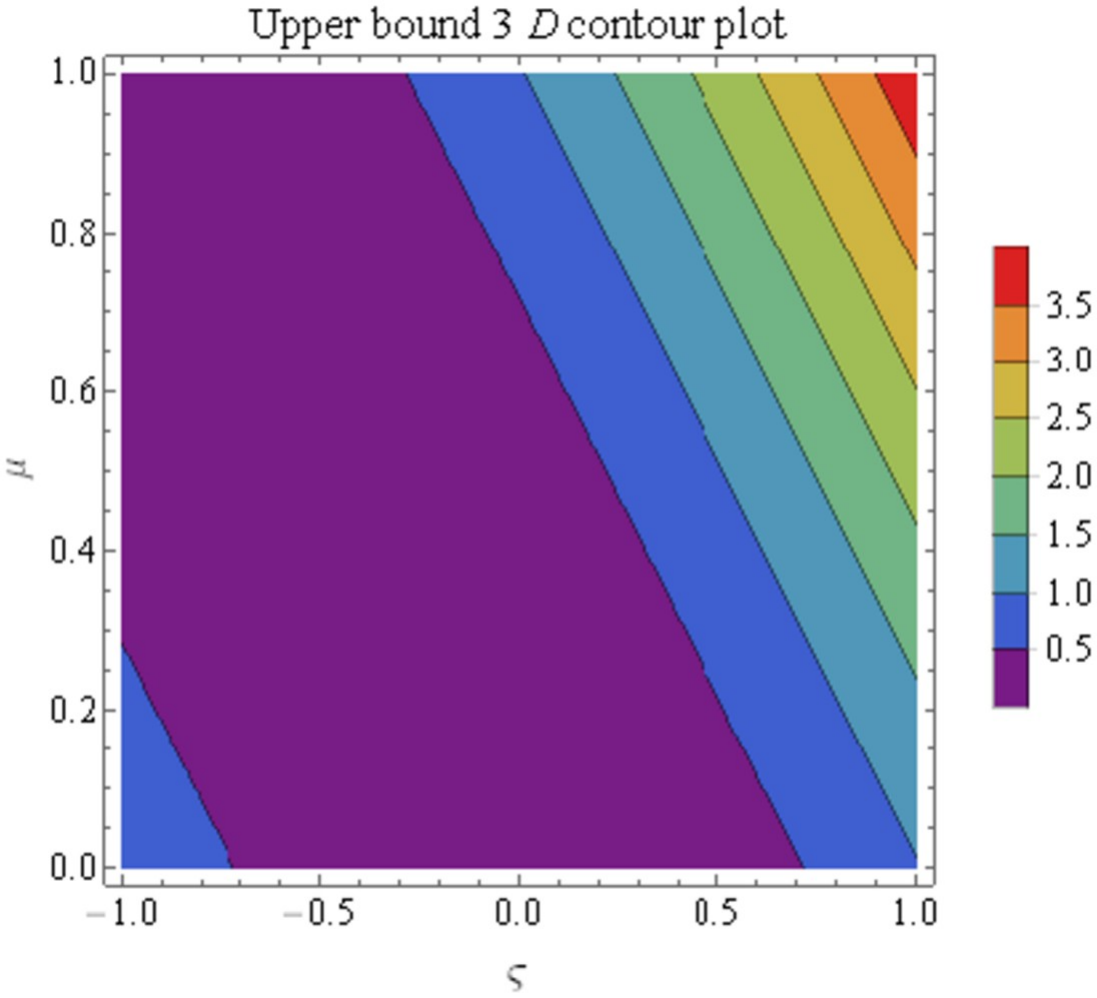
Contour structure of $\bar{\vartheta }\left(\varsigma ,\mu ,\tau \right)$ upon *α* = 1.

**Fig 29:**
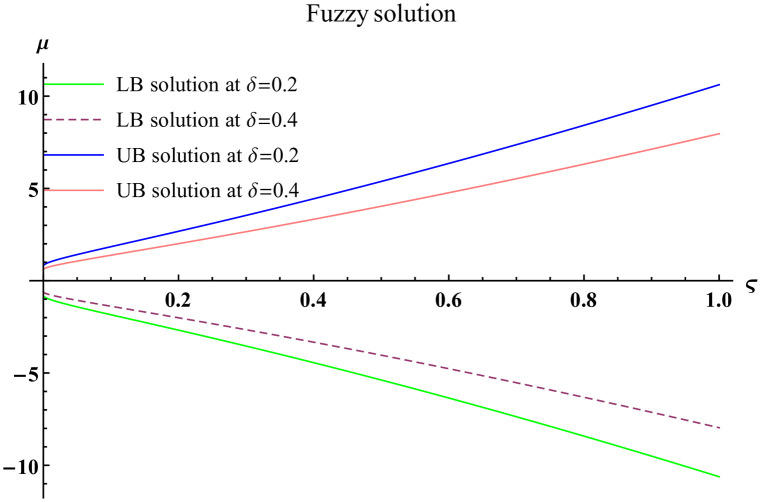
2D fuzzy plot of $\tilde{\vartheta }\left(\varsigma ,\mu ,\tau \right)$ upon *α* = 0.75.

**Fig 30:**
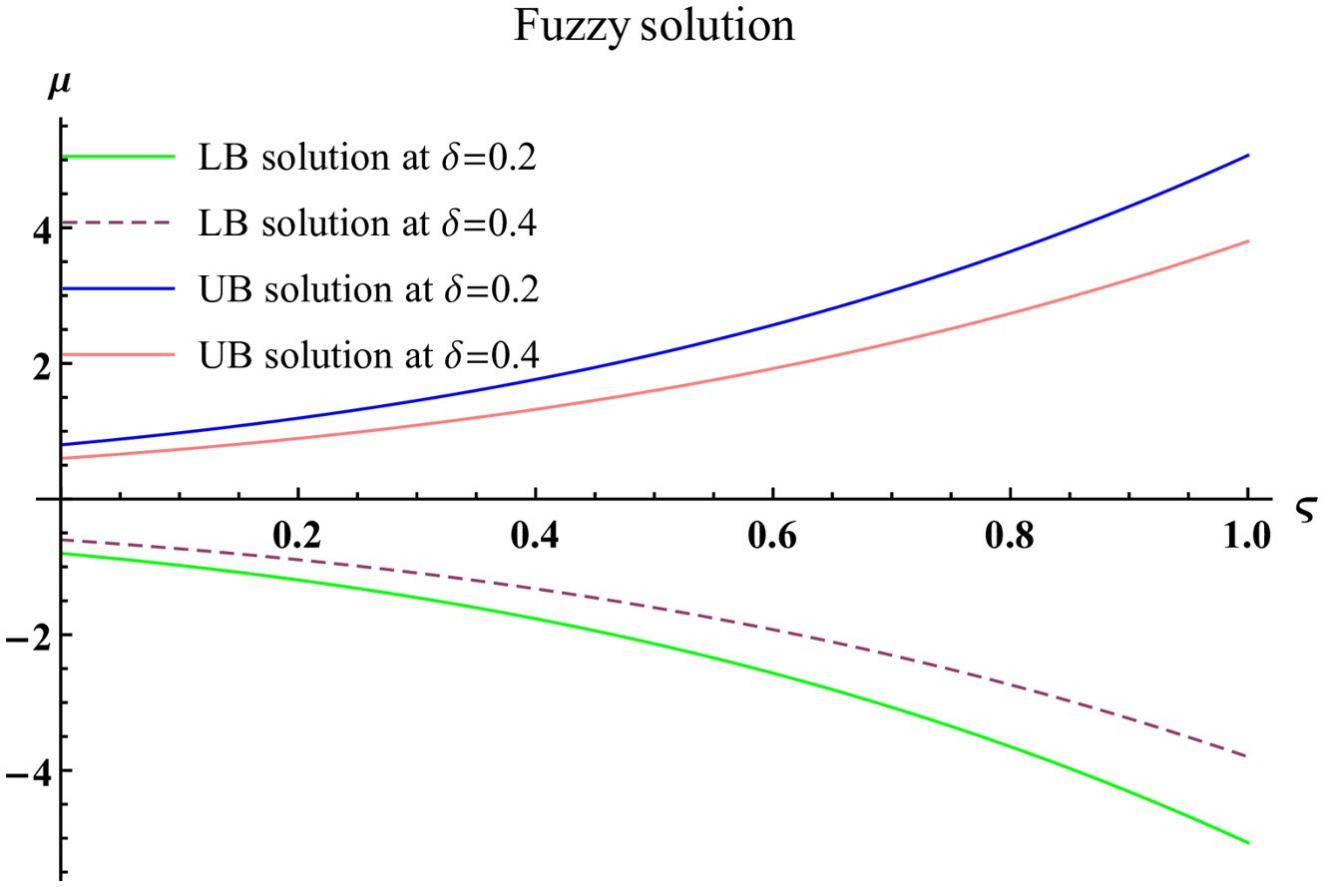
2D fuzzy plot of $\tilde{\vartheta }\left(\varsigma ,\mu ,\tau \right)$ upon *α* = 1.

We explain the physical nature of Example (4.3) in its several graphical structures. We include surface and contour plots that demonstrate the physical nature of the fuzzy heat fractional problem for for lower bound approximation. Figs 21 and 22 state the 3D graphical depictions of lower bound approximation at different coordinates of parameters at *τ* = 0.1 with −2 ≤ *ς* ≤ 2 and −2 ≤ *μ* ≤ 2 for *α* = 0.75 and *α* = 1 respectively. Similarly, Figs 23 and 24 show the contour plots of lower bound approximation at different coordinates of parameters at *τ* = 0.1 with −1 ≤ *ς* ≤ 1 and 0 ≤ *μ* ≤ 1 for *α* = 0.75 and *α* = 1 respectively. We include surface and contour plots that demonstrate the physical nature of the fuzzy heat fractional problem for upper bound approximation. Figs 25 and 26 state the 3D graphical depictions of upper bound approximation at different coordinates of parameters at *τ* = 0.1 with −2 ≤ *ς* ≤ 2 and −1 ≤ *μ* ≤ 1 for *α* = 0.75 and *α* = 1 respectively. Similarly, Figs 27 and 28 show the contour plots of upper bound approximation at different coordinates of parameters at *τ* = 0.1 with −1 ≤ *ς* ≤ 1 and 0 ≤ *μ* ≤ 1 for *α* = 0.75 and *α* = 1 respectively. In Figs 29 and 30, we demonstrate a 2D fuzzy structure for lower and upper bound approximation across the parameters *δ* = 0.2 and *δ* = 0.4. Our graphical structures of 3D and 2D show that the suggested scheme is valid and consistent for fuzzy fractional scenarios.

## 5 Conclusion

This paper introduces a novel scheme that combines the Sawi transform operator and the residual power series to address fuzzy heat time-fractional two-dimensional models with an external source variable. The advantage of this novel scheme is its capability to decrease the mathematical work required for determining the result in a power series structure whereas the unknown components are determined through a series of algebraic procedures. The suggested method is employed for analyzing three distinct physical models, and its validity has been demonstrated in effectively handling fractional nonlinear equations with both high precision and simplified calculation processes. The numerical findings indicate that the approximating results closely align with each other and have complete affirm with the precise results for *α* = 1. We examine the behavior of the derived results both numerically and graphically for a particular range of *α* values. One major benefit of SRPSS is its ability to derive series coefficients with few calculations, as it relies on the concept of limits instead of fractional derivatives. This indicates that the suggested scheme is suitable and extremely effective for generating approximate and analytical solutions to fuzzy fractional problems that occur in engineering and applied physics. We intend to expand the application of the Sawi residual power series scheme to address physical problems with more complex three-dimensional models in our upcoming work.

## Supporting information

S1 File (DOCX)
