# Correction: Numerical investigation of two-dimensional fuzzy fractional heat problem with an external source variable

**DOI:** 10.1371/journal.pone.0307846

**Published:** 2024-07-22

**Authors:** Muhammad Nadeem, Saad H. Alotaibi, Fawziah M. Alotaibi, Yahya Alsayaad

There is an error in acknowledgement section. The correct acknowledgement section is: The authors extend their appreciation to Taif University, Saudi Arabia, for supporting this work.
