# Supplementary material for: Numerical investigation of two-dimensional fuzzy fractional heat problem with an external source variable
Source: PLoS One. 2024 Jun 21;19(6):e0304871. doi: 10.1371/journal.pone.0304871 (PMC11192405; doi:10.1371/journal.pone.0304871)
Supplement: S1 File — (DOCX) [file pone.0304871.s001.docx]

**Minimal Data Set**

| **Figures** | **Subfigure** | **$\delta$** | **$\varsigma$** | **$\mu$** | **$\tau$** | **$\alpha=0.5$** | **Solution** | **$\alpha=1$** | **Solution** |
| --- | --- | --- | --- | --- | --- | --- | --- | --- | --- |
| **Figure 1** | (a) | 0.2 | 0.25 | 0.25 | 0.5 | 0.5 | -2.53455 | 1 | -1.11197 |
|  | (b) | 0.2 | 0.50 | 0.50 | 0.5 | 0.5 | -1.53728 | 1 | -0.674446 |
|  | (c) | 0.2 | 0.75 | 0.75 | 0.5 | 0.5 | -0.93240 | 1 | -0.409072 |
|  | (d) | 0.2 | 1 | 1 | 0.5 | 0.5 | -0.56553 | 1 | -0.248115 |
| **Figure 2** | (a) | 0.8 | 0.25 | 0.25 | 0.5 | 0.5 | -0.63363 | 1 | -0.277993 |
|  | (b) | 0.8 | 0.50 | 0.50 | 0.5 | 0.5 | -0.38432 | 1 | -0.168611 |
|  | (c) | 0.8 | 0.75 | 0.75 | 0.5 | 0.5 | -0.23310 | 1 | -0.102268 |
|  | (d) | 0.8 | 1 | 1 | 0.5 | 0.5 | -0.14138 | 1 | -0.0620287 |
| **Figure 3** | (a) | 0.4 | 0.25 | 0.25 | 0.3 | 0.5 | -1.28279 | 1 | -0.611747 |
|  | (b) | 0.6 | 0.50 | 0.50 | 0.3 | 0.5 | -0.51870 | 1 | -0.247362 |
| **Figure 4** | (a) | 0.2 | 0.25 | 0.25 | 0.5 | 0.5 | 12.8079 | 1 | 4.46379 |
|  | (b) | 0.2 | 0.50 | 0.50 | 0.5 | 0.5 | 1.56852 | 1 | 5.46656 |
|  | (c) | 0.2 | 0.75 | 0.75 | 0.5 | 0.5 | -12.8079 | 1 | -4.46379 |
|  | (d) | 0.2 | 1 | 1 | 0.5 | 0.5 | 0 | 1 | 0 |
| **Figure 5** | (a) | 0.8 | 0.25 | 0.25 | 0.5 | 0.5 | 3.20198 | 1 | 1.11595 |
|  | (b) | 0.8 | 0.50 | 0.50 | 0.5 | 0.5 | 3.92129 | 1 | 1.36664 |
|  | (c) | 0.8 | 0.75 | 0.75 | 0.5 | 0.5 | -3.20198 | 1 | -1.11595 |
|  | (d) | 0.8 | 1 | 1 | 0.5 | 0.5 | 0 | 1 | 0 |
| **Figure 6** | (a) | 0.4 | 0.25 | 0.25 | 0.3 | 0.5 | 5.46945 | 1 | 2.10032 |
|  | (b) | 0.6 | 0.50 | 0.50 | 0.3 | 0.5 | 4.46543 | 1 | 1.71477 |
| **Figure 7** | (a) | 0.2 | 0.25 | 0.25 | 0.5 | 0.5 | -1.04469 | 1 | -0.458333 |
|  | (b) | 0.2 | 0.50 | 0.50 | 0.5 | 0.5 | -4.17877 | 1 | -1.83333 |
|  | (c) | 0.2 | 0.75 | 0.75 | 0.5 | 0.5 | -9.40223 | 1 | -4.125 |
|  | (d) | 0.2 | 1 | 1 | 0.5 | 0.5 | -16.7151 | 1 | -7.33333 |
| **Figure 8** | (a) | 0.8 | 0.25 | 0.25 | 0.5 | 0.5 | -0.26117 | 1 | -0.114583 |
|  | (b) | 0.8 | 0.50 | 0.50 | 0.5 | 0.5 | -1.04469 | 1 | -0.458333 |
|  | (c) | 0.8 | 0.75 | 0.75 | 0.5 | 0.5 | -2.35056 | 1 | -1.03125 |
|  | (d) | 0.8 | 1 | 1 | 0.5 | 0.5 | -4.17877 | 1 | -1.83333 |
| **Figure 9** | (a) | 0.4 | 0.25 | 0.25 | 0.3 | 0.5 | -0.52874 | 1 | -0.25215 |
|  | (b) | 0.6 | 0.50 | 0.50 | 0.3 | 0.5 | -1.40998 | 1 | -0.6724 |
